# *In silico* assessment of genetic variation in *KCNA5* reveals multiple mechanisms of human atrial arrhythmogenesis

**DOI:** 10.1371/journal.pcbi.1005587

**Published:** 2017-06-16

**Authors:** Michael A. Colman, Haibo Ni, Bo Liang, Nicole Schmitt, Henggui Zhang

**Affiliations:** 1 Biological Physics Group, School of Physics and Astronomy, University of Manchester, Manchester, United Kingdom; 2 School of Biomedical Sciences, Faculty of Biological Sciences, University of Leeds, Leeds, United Kingdom; 3 Department of Biomedical Sciences, Faculty of Health and Medical Sciences, University of Copenhagen, Copenhagen, Denmark; 4 School of Computer Science and Technology, Harbin Institute of Technology, Harbin, China; Universiteit Gent, BELGIUM

## Abstract

A recent experimental study investigating patients with lone atrial fibrillation identified six novel mutations in the *KCNA5* gene. The mutants exhibited both gain- and loss-of-function of the atrial specific ultra-rapid delayed rectifier K^+^ current, *I*_Kur_. The aim of this study is to elucidate and quantify the functional impact of these *KCNA5* mutations on atrial electrical activity. A multi-scale model of the human atria was updated to incorporate detailed experimental data on *I*_Kur_ from both wild-type and mutants. The effects of the mutations on human atrial action potential and rate dependence were investigated at the cellular level. In tissue, we assessed the effects of the mutations on the vulnerability to unidirectional conduction patterns and dynamics of re-entrant excitation waves. Gain-of-function mutations shortened the action potential duration in single cells, and stabilised and accelerated re-entrant excitation in tissue. Loss-of-function mutations had heterogeneous effects on action potential duration and promoted early-after-depolarisations following beta-adrenergic stimulation. In the tissue model, loss-of-function mutations facilitated breakdown of excitation waves at more physiological excitation rates than the wild-type, and the generation of early-after-depolarisations promoted unidirectional patterns of excitation. Gain- and loss-of-function *I*_Kur_ mutations produced multiple mechanisms of atrial arrhythmogenesis, with significant differences between the two groups of mutations. This study provides new insights into understanding the mechanisms by which mutant *I*_Kur_ contributes to atrial arrhythmias. In addition, as *I*_Kur_ is an atrial-specific channel and a number of *I*_Kur_-selective blockers have been developed as anti-AF agents, this study also helps to understand some contradictory results on both pro- and anti-arrhythmic effects of blocking *I*_Kur_.

## Author summary

In a recent study, six mutations resulting in either gain-of-function or loss-of-function in the ultra-rapid delayed rectifier potassium current *I*_Kur_, were identified to be associated with atrial fibrillation (AF). However, the causative link between the mutant *I*_Kur_ (either gain- or loss-of-function) and AF genesis, especially the difference and similarity between the two mutant groups, has not been elucidated. In our study, we used multiscale computational models to investigate the mechanism of arrhythmogenesis mediated by the two groups of mutations. The results suggest that the gain-of-function mutations shortened atrial action potential duration, stabilised and accelerated re-entrant excitation waves in tissue; the loss-of-function mutation promoted early-after-depolarisations following beta-adrenergic stimulation and thus wave breaks in tissue. We show these two groups of mutants carrying *I*_Kur_ produced multiple mechanisms of atrial arrhythmogenesis, with significant differences between the two groups. Our study provides new insights into understanding the mechanisms by which mutant *I*_Kur_ contributes to atrial arrhythmias. In addition, as *I*_Kur_ is an atrial-specific channel and a number of *I*_Kur_-selective blockers have been developed as anti-AF agents, this study also helps to understand some contradictory results on both pro- and anti-arrhythmic effects of blocking *I*_Kur_.

## Introduction

Mutations in genes encoding the proteins involved in cardiac electrophysiology can result in alterations of the electrical action potential (AP) and tissue excitability, which may affect cardiac output and predispose to arrhythmias, such as atrial fibrillation (AF) [1,2].

AF, the world’s most common cardiac arrhythmia [3], is characterised by complex and rapid electrical activation of the upper chambers of the heart, impairing cardiac function and significantly increasing the risk of heart failure, stroke and death [4]. Electrical activity driving AF is conjectured to be underlain by ectopic pacing (abnormal spontaneous activity), re-entrant excitation (in the form of scroll waves or wavelets), or an interplay between both [5,6].

AF can arise within the context of other heart conditions, such as heart failure, and is in general associated with large-scale and multi-faceted electrical and structural remodelling [7–9], but may also occur in the presence of individual genetic mutations in the otherwise normal heart (referred to as ‘lone’ or ‘familial’ AF). Understanding the pro-arrhythmic role of genetic variation is an essential part of the wider effort to understand and treat the cardiac disorder.

The ultra-rapid potassium current (*I*_Kur_) plays an important role in repolarisation of the atrial action potential in human and other animals [2,10]. Alterations to the biophysical properties of *I*_Kur_ are associated with incidences of AF [2,8]. A recent study analysing patients with lone AF has identified six mutations in the human *KCNA5* gene, which encodes the Kv1.5 channel carrying *I*_Kur_ [2]. Of the six mutations, three resulted in a gain-of-function (D322H, A305T, E48G) and three in a loss-of-function (P488S, Y155C, D469E) of the current. All mutations resulted in alterations of the channel’s maximum current density and kinetics [2], which is likely to manifest as modifications to AP morphology and duration.

AP morphology and duration are critical factors determining complex electrical wave dynamics in tissue, such as those observed during AF. Previous studies have demonstrated that AP duration (APD) abbreviation plays an important role in sustaining re-entrant circuits [11–13]. Moreover, large gradients in AP morphology and duration across different regions of the atria can promote wave-breaks, leading to unidirectional conduction patterns that can develop into self-perpetuating re-entrant excitation [13–15]. *I*_Kur_ is active during the plateau and early repolarisation phase of the human atrial AP, affecting AP morphology and duration and thus providing the potential to perpetuate and facilitate atrial arrhythmias.

Recent experimental and modelling studies have also demonstrated an important role for *I*_Kur_ in the incidence of early-after-depolarisations (EADs) during beta-adrenergic stimulation (sympathetic nervous response) in isolated cells [16,17]. After-depolarisations are generally linked with arrhythmogenic mechanisms [18–20] and thus mutations which affect *I*_Kur_ dynamics may also mediate arrhythmogenesis through the promotion of EADs.

Diversity in *I*_Kur_ kinetics associated with genetic variation of *KCNA5* therefore presents the possibility to facilitate atrial arrhythmogenesis through different mechanisms. Determining these mechanisms is the aim of the present study. This is achieved through integrating experimental data on diversity in *I*_*Kur*_ kinetics into a multi-scale computational model of the human atria, used to elucidate the functional impact of the six identified mutations on electrical activity at the cellular and tissue scale. In order to exclude possible model-dependent results and conclusion, three contemporary mathematical models of human atrial electrophysiology [13,17,21] were implemented in this study.

## Results

### Gain-of-function mutations

**Fig 1:**
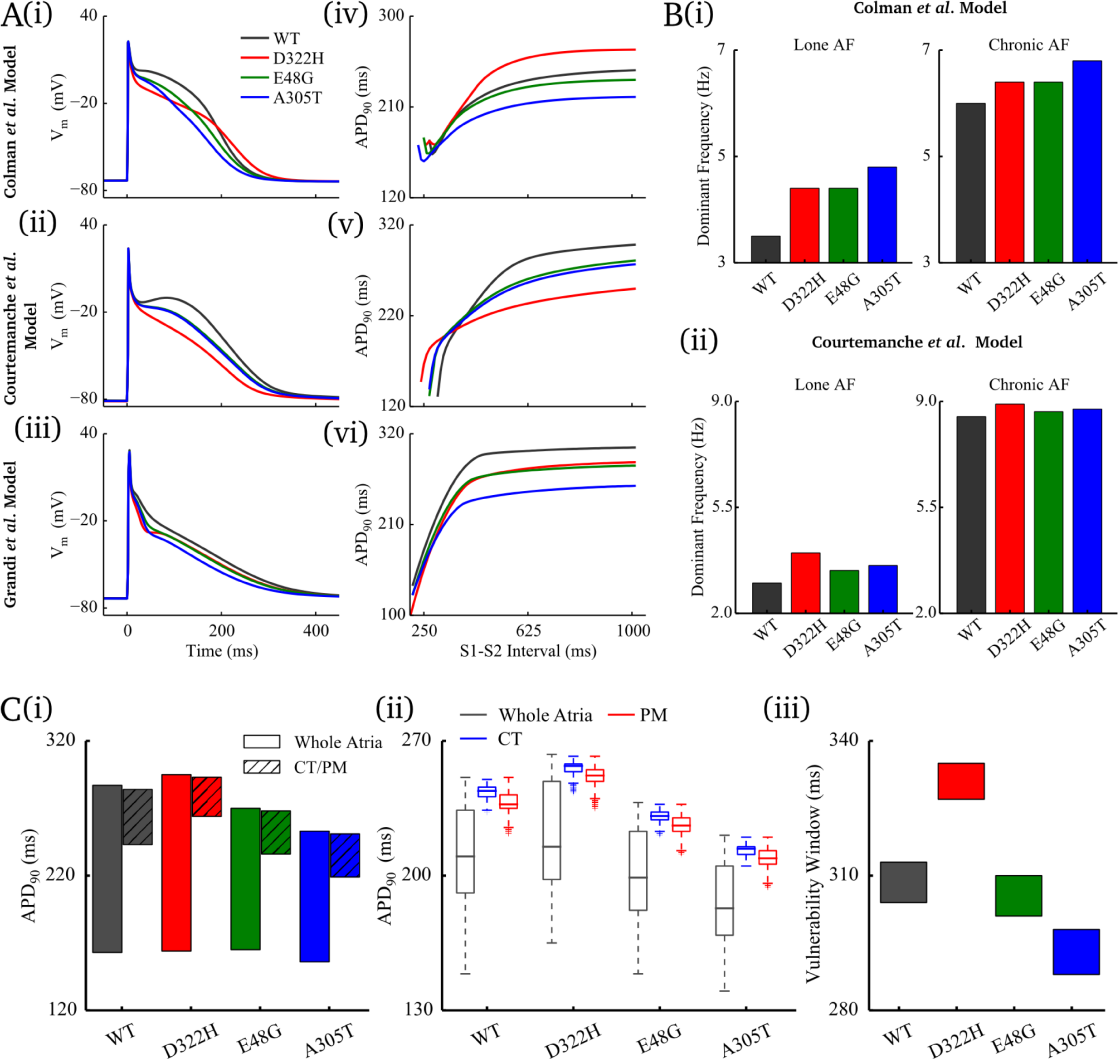
Effects of gain-of-function *KCNA5* mutations. **A** Effects of the mutations on the AP (i-iii) and APD-restitution (iv-vi) properties of human atrial myocytes elicited by updated *Colman et al*. model, *Courtemanche et al*. model and *Grandi et al*. model. **B** Effects of the mutations on the maximum sustained dominant frequency of excitation waves under the lone AF and chronic AF conditions using (i) *Colman et al*. model and (ii) *Courtemanche et al*. model. **C** Effects of the mutations on APD heterogeneity and tissue vulnerability window at the CT/PM junction in *Colman et al*. model. APD distribution among regional cells of whole atria and CT/PM for in (i) isolated single cells and (ii) in coupled tissue; the APD distribution in tissue is shown in boxplots with outlier limits of 1.5×IQR (interquartile range). (iii) Temporal vulnerability window to propagation wave break at the CT/PM junction.

All gain-of-function mutations had a marked effect on AP morphology and duration (Fig 1A, detailed description in S1 Text). In lone AF, the notch and plateau potentials and APD_30_ were reduced, and APD_90_ was in general shortened; the APD restitution curves were flattened. With the inclusion of chronic AF (cAF) electrical remodelling, all mutations shortened the APD_90_ in all models (S2 Text).

In simulations using the *Colman et al*. [13] and *Courtemanche et al*. [21] single cell models mapped onto the 3D reconstruction of human atrial anatomy, all three gain-of-function mutations promoted stable, higher frequency re-entrant scroll waves compared to the WT in all models (Fig 1B, Figure A in S3 Text). Re-entry could also be sustained at larger values of the diffusion coefficient (***D***), closer to control, indicating an increased susceptibility to re-entry in the structurally normal atria, or atria with a normal electrical coupling (Table A in S3 Text). cAF remodelling accelerated and thus stabilised re-entrant excitation in all mutations and WT compared to the lone AF condition and in all mutations compared to the WT (Fig 1Bi,ii).

The extent of APD_90_ variation across electrically heterogeneous regions was in general reduced by these mutations compared to the WT; APD_90_ variation was also reduced in coupled tissue compared to single cells for all conditions (Fig 1Ci,ii). However, at the heterogeneous junction of crista terminalis/pectinate muscle (CT/PM), the in-tissue APD_90_ difference between the two regions was only affected by a small degree by the mutations. Correspondingly, the mutations had only a small effect on the width of the vulnerability windows (VWs) under S1S2 pacing, but caused a marked shift to the excitation cycle-lengths of S2 over which the VWs were observed (Fig 1Ciii, S4 Text).

### Loss-of-function mutations

**Fig 2:**
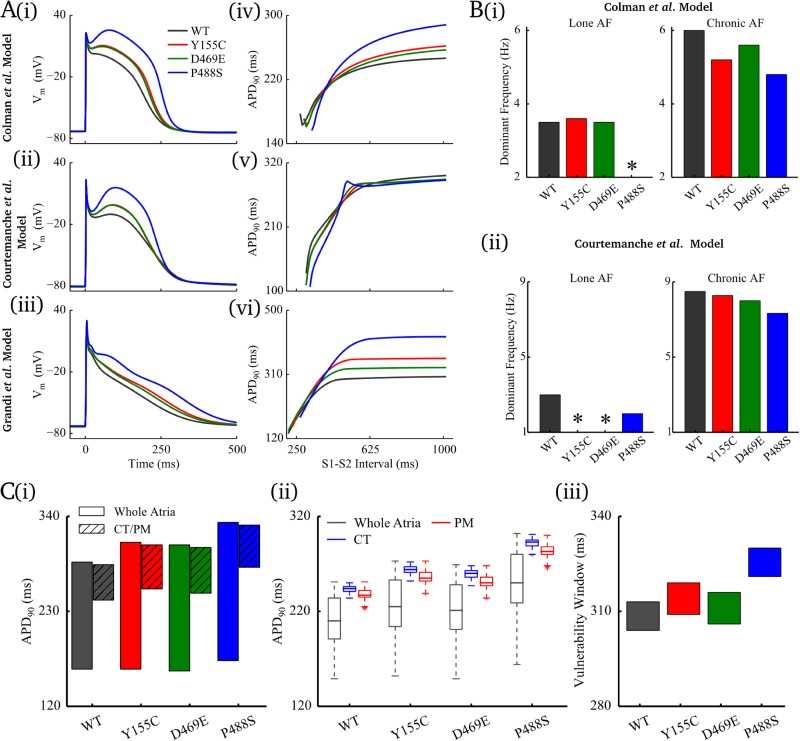
Effects of loss-of-function mutations. **A** Effects of the mutations on AP (i-iii) and APD-restitution (iv-vi) properties of human atrial myocytes elicited by updated *Colman et al*. model, *Courtemanche et al*. model and *Grandi et al*. model. **B** Effects of the mutations on the maximum sustained dominant frequency of excitation waves in the lone AF and chronic AF conditions using (i) *Colman et al*. model and (ii) *Courtemanche et al*. model. **C** Effects of *KCNA5* mutations on APD heterogeneity and tissue vulnerability window at the CT/PM junction in *Colman et al*. model. APD distribution among regional cells of whole atria and CT/PM for in (i) isolated single cells and (ii) in coupled tissue; the APD distribution in tissue is shown in boxplots with outlier limits of 1.5×IQR (interquartile range). (iii) Temporal vulnerability window to wave propagation break at the CT/PM junction. In panel B, * indicates cases in which re-entrant waves could not be sustained.

Loss-of-function mutations markedly altered AP morphology, but had a less marked and consistent effect on APD_90_ among the three models: enhanced plateau/dome phase of the AP and thus APD_30_ was observed in all mutations, but secondary effects on terminal repolarisation led to variations in modulation of APD_90_ (Fig 2A and discussed further in S1 Text); in different models and mutations, the effects on APD_90_ ranged from substantial prolongation to slight shortening (Fig 2A). Note that in previous experimental studies, blocking *I*_Kur_ either resulted in an increase [7] or decrease [22] in APD of human atrial cells depending on the baseline morphology of the atrial AP.

Under cAF conditions, consistent effects were observed between the individual mutations in the three models (S2 Text): all mutations elevated AP-plateau and prolonged APD_90_.

In 3D organ-scale simulations, the mutations presented inconsistent effects on the stability of re-entrant excitation: D469E and Y155C had very little effect on the dominant frequency in the *Colman et al*. model (Fig 2Bi), whereas re-entry could not be sustained in P488S (transient re-entry had a lifetime of less than a second, even in the severe remodelling condition), but in the *Courtemanche et al*. model, the presence of alternans with D469E and Y155C destabilised the re-entrant waves, leading to termination of rotors (Fig 2Bii, Figure B in S3 Text).

Analogously to gain-of-function mutations, loss-of-function mutations increased APD_90_ heterogeneity across the atria, but had a relatively small effect on the heterogeneity at the CT/PM junction and correspondingly the size of the VWs, whereas the shift in temporal window of S2 over which wave breaks were observed (shifted to longer values) was more substantial (Fig 2C, S4 Text).

### Beta-adrenergic stimulation

**Fig 3:**
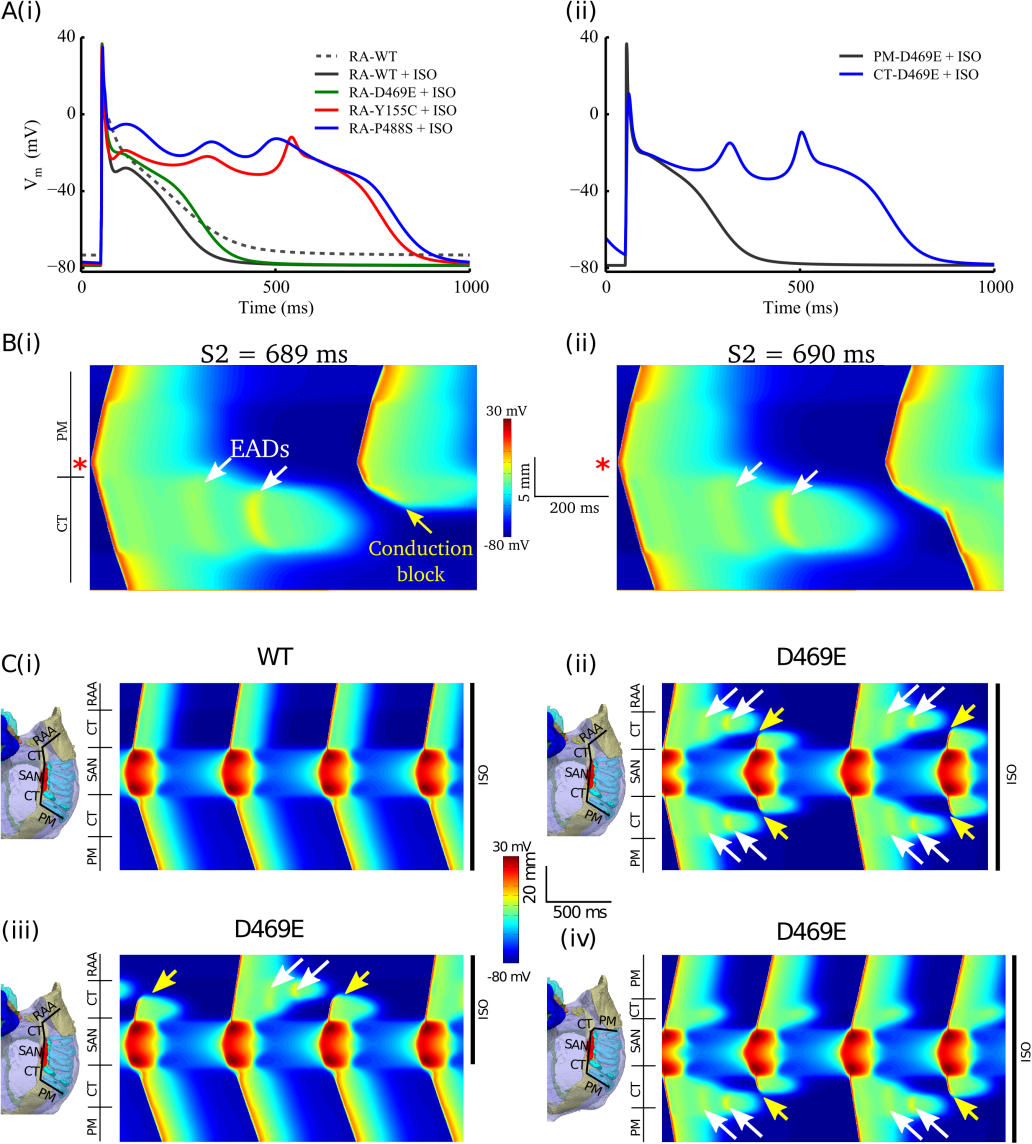
*KCNA5* loss-of-function mutations induced EADs following the beta-adrenergic stimulation. **A**(i) In the presence of ISO, EADs were produced by Y155C and P488S, but not in WT or D469E in RA; (ii) EADs were induced by D469E in CT but not in PM. **B** Under uniform application of ISO in a 1D strand model with D469E, EADs in the CT but not PM induced conduction block at an S2 of 689 ms (i) and success at 690 ms (ii). **C** 1D conduction patterns under mutations and application of ISO in various configurations. On the left of each panel is a breakdown of the regions in the 1D model and an illustration of the anatomical conduction pathway to which they correspond; on the right is the regions of tissue to which ISO was applied. (i) Regular conduction pattern under WT and uniform ISO application; (ii) Under uniform application of ISO, D469E led to alternating bidirectional conduction block due to EADs in the CT; (iii) D469E and non-uniform ISO can lead to unidirectional conduction block, resulting from EADs in the CT regions with ISO; (iv) Unidirectional block can also be attained through a different pathway to the PM, in which the CT on one side of the SAN is of insufficient extent to develop significant EADs. In the simulations effects of beta-adrenergic stimulation was modelled by simulated application of ISO (1 μM).

The application of Isoprenaline (ISO, 1μM), replicating beta-adrenergic stimulation, had no arrhythmogenic impact on myocytes with gain-of-function mutations or WT *I*_Kur_ (Figure A in S5 Text). However, for the loss-of-function mutants pronounced EADs were observed in simulations using the *Grandi et al*. model (Fig 3A, Figure B in S5 Text). In simulations across regional cell models, the CT was more susceptible to the development of EADs than the PM and RA (Fig 3A) due to its larger expression of *I*_*CaL*_ [13,14]: more substantial EADs were observed in the CT for Y155C and P488S, whereas D469E led to EADs in the CT but none in the PM or RA.

Implications of observed EADs on tissue excitation patterns were evaluated in a 1D strand of human atrial cell models. In these simulations, substantial EADs were observed (Fig 3B). The increased susceptibility of the CT to EADs compared to the surrounding atria (PM, RA) was also observed. Using a 1D strand model of the CT and PMs only, EADs in the CT (for the case of D469E) could not cause a premature propagating excitation wave (*i*.*e*., focal activity) into the adjacent PMs or RA (which did not exhibit EADs) under either rapid or slow S1 pacing.

An S1S2 pacing protocol was used to evaluate the effect of EAD-mediated APD prolongation on the VW at the heterogeneous CT/PM junction. For D469E plus ISO, a VW with a substantial width (313 ms) and an upper limit at a long cycle length (689 ms) (Fig 3B) was observed–significantly larger than WT or VWs computed in the absence of EADs. VWs of a similar size were observed in all cases of heterogeneous EAD production resulting from a heterogeneous application of ISO (D469E at the CT/PM junction; non-uniform ISO distributions for loss-of-function mutations).

1D tissue simulations including a spontaneously active sinoatrial node (SAN) with various regional and ISO configurations (Fig 3C) indicated that production of EADs in the CT could interrupt conduction patterns of excitation emanating from the SAN (Fig 3C): significant EADs at both exit pathways from the SAN to the CT caused “skipped beats” through bi-directional block; EADs in only one pathway (non-uniform ISO distribution; short pathway to the PM) resulted in unidirectional conduction block. Applied focal pacing was therefore not necessary to induce potentially arrhythmic conduction patterns.

## Discussion

In this study, the impact of six mutations in *KCNA5* on human atrial electrical function was investigated *in silico*, which elucidates and clarifies the arrhythmogenic effect of the six identified *KCNA5* mutations, demonstrating a causal link between the mutations and incidence of arrhythmia—a link which is distinctly different for the two groups of mutations. The opposing effects on the AP due to gain- and loss- of-function mutations, and their respective mechanistic pathways to induction of arrhythmia, indicate that pharmacological intervention to treat the condition may need to be considered specifically for the genotype and the resulting phenotype. The presented results have implications that can be extrapolated beyond the specific mutations, providing general insight into the role of *I*_Kur_ kinetics in atrial electrical function, further to previous modelling [15,23–25] or experimental [26–28] studies, which primarily have focused on anti-arrhythmic properties of *I*_Kur_ block/down-regulation alone.

### Arrhythmia mechanisms of gain-of-function mutations

Gain-of-function mutations promoted the initiation and sustenance of arrhythmic re-entrant excitation through shortening of the action potential duration and therefore excitation wave-length [13].

In single cells, the increased activity of the repolarising current during phase-2 of the AP led to a significant reduction of the plateau potential. In 8 out of 9 cases this directly contributed to shortened terminal repolarisation and refractory period and flattening of the APD restitution curve, resulting in shorter refractory period across the range of pre-pacing cycle lengths and thus promoting sustained re-entry [6]. The exception is D322H in the *Colman et al*. model, which led to APD_90_ prolongation in single cells (Figure A in S1 Text). However, even in this condition, with the additional load of electrotonic coupling (which compensates for the loss of terminal repolarising currents *I*_Ks_ and *I*_Kr_−discussed further in S3 Text), the in-tissue APD was nonetheless shortened at the rapid rates associated with ectopic pacing and re-entry (Figure A in S3 Text), leading to promotion of sustained re-entry.

Simulations in this study did not highlight a direct mechanism by which these mutations promoted the initiation of re-entry (i.e. the VWs were not increased). However, other studies have demonstrated that the presence of increased fibrotic lesions [29,30] can lead to conduction wave-breaks and the development of re-entry. In these conditions, it is well established that a shortening of the wavelength enhances the probability of a wave-break developing into self-perpetuating re-entry [5,13], and in the present study we have demonstrated that re-entry is stabilised in these mutations once initiated, and that stable re-entrant waves could be sustained in atria with less severe remodelling in the gap-junctions / structure (Table A in S3 Text). Therefore, AP shortening due to increased activity of *I*_Kur_ can promote both the initiation and maintenance of re-entry, associated with AF, when combined with other factors (such as ectopic pacing and fibrosis).

### Arrhythmia mechanisms of loss-of-function mutations

Loss-of-function mutations enhanced the susceptibility of atrial tissue to the induction of arrhythmia through a positive shift in the cycle lengths over which the VWs were observed and enhanced vulnerability to EADs, but did not promote the sustenance of re-entrant excitation. Enhanced vulnerability to the induction of wave-breaks may be mechanistically linked to the development and subsequent breakdown of re-entry.

In single cell, the loss of repolarising current during phase 2 of the AP elevated the AP-plateau. This could lead directly to a prolongation of the APD_90_, but through secondary effects on other currents, the APD_90_ could be unchanged or slightly shortened compared to the WT (S1 Text). This inconsistency in the modulation of the refractory period led to variable effects on the maintenance of re-entry.

This loss of repolarising current and elevation of the AP plateau also underlies enhanced susceptibility to EADs when combined with ISO: the increased activity of *I*_CaL_ is no longer counteracted by sufficient repolarising *I*_Kur_, leading to the possibility of re-activation of *I*_CaL_ driving an EAD (S5 Text). This mechanism has been previously observed in both experimental and modelling studies in which *I*_Kur_ is pharmacologically blocked [16,17]. Whereas our simulations did not demonstrate focal activity resulting from EADs (consistent with [19,31] which demonstrated that phase 3 EADs, not phase 2 as observed in the present study, are necessary for the development of focal activity), spatially heterogeneous production of EADs led to significant in-tissue APD gradients and substantial VWs.

EADs in the CT were also observed to block excitation originating from the SAN, providing a potential mechanism for the development of re-entry independent of ectopic pacing. Thus, these mutations can promote the development of asymmetric conduction patterns which are a precursor to re-entry, and promote both the initiation and recurrence of AF, when combined with factors which promote sustained re-entry, such as connexin and structural remodelling or further electrical remodelling. We note that these latter factors are likely necessary to fully account for the incidence of AF in these patients.

### The role of *I*_Kur_ in action potential morphology

Our results indicate a complex role for *I*_Kur_ kinetics and magnitude in atrial AP morphology, which differed in variant models. Analysis demonstrated that the interplay between the extent of activation of *I*_Ks_ and *I*_Kr_ and deactivation kinetics of *I*_Kur_ determined the dependence of APD_90_ on *I*_Kur_. Details are provided in S1 Text, but briefly: elevation or reduction of the AP plateau led to enhanced or reduced activity in *I*_Ks_ and *I*_Kr_, respectively. The impact of these secondary effects is determined by WT AP morphology, the extent of modulation directly by *I*_Kur_, the rapidity of *I*_Kur_ deactivation, and the formulations of *I*_Ks_ and *I*_Kr_ themselves, leading to different behaviour in different models and mutations. Due to inter-subject variations and inter-cellular variations from the same patient, this non-linear behaviour may be clinically relevant. Consistency between the models in cAF conditions results from the similar triangular AP morphology exhibited by all models, wherein cAF remodelling dominates AF modulation.

### Use of multiple, independent models of human atrial electrophysiology

Over the years, a number of mathematical models have been developed with different formulations for ionic currents as well as intracellular calcium handling system based on different experimental datasets [13,17,21,32]. Consequently these models produce variable electrophysiological properties which represent the significant variations observed experimentally, including three distinct AP profiles in human atrial myocytes [33]. In this study, three contemporary cell models were implemented to ensure that simulation results and conclusions are model-independent. Whereas the major conclusions were drawn from observations common to all models, some specific behaviour did vary among the implemented models and warrants discussion.

Variation between the models and mutations regarding single cell is explained by the analysis of the non-linear role of *I*_Kur_ in AP morphology (S1 Text). In this instance, use of multiple models was essential in revealing this property and directly leads to our conclusions regarding inconsistency in loss-of-function mutations modulating stable re-entry.

The genesis of EADs, and consequently the role in generating unidirectional conduction block, was only observed in the *Grandi* et al. cell model. Whereas this behaviour could also be induced in further simulations using the *Nygren et al*. model [34] (Figure C in S5 Text) it could not be in the *Courtemanche et al*. and *Colman et al*. models (S5 Text). The authors note that production of EADs due to ISO and a loss of *I*_Kur_ is experimentally observed [16] and natively reproduced only by the *Grandi et al*. model, and it follows that such activity could result from a loss-in-function mutation as well as a pharmacological blockade. Therefore it is likely that an enhanced susceptibility to EADs due to loss-of-function mutations may be observed clinically and warrants further investigation. Furthermore, our analysis of the effect of EADs on atrial conduction patterns provides general mechanistic insight, beyond that specific to the mutations studied, to the potentially arrhythmic role of EADs.

### Clinical relevance

Whereas the present study provides insight into the link between the identified mutations in *KCNA5* and the incidence of AF, *I*_Kur_ is also of more general interest as a target for AF management: it is atrial specific [2] and thus provides an attractive prospect for pharmacological intervention because it can be targeted without adverse effects on the ventricles. Despite this, the efficacy of *I*_Kur_ blockers to terminate AF has been shown to be controversial in modelling [15,24] and experimental [26] studies.

Our results add insight to the on-going discussion: blocking *I*_Kur_ may help to terminate re-entry, but also increases tissue’s vulnerability to AF initiation. Furthermore, dependent on baseline AP morphology, blocking *I*_Kur_ may shorten the APD, facilitating sustained re-entry. Moreover, for paroxysmal AF of which AP parameters could be unaltered [35], blocking *I*_Kur_ may result in pro-arrhythmic consequences, such as EADs.

### Limitations

Limitations of the cellular and 3D models, as well as experimental data acquisition, are discussed elsewhere [2,13,14]. Here, only limitations associated with the cellular and tissue modelling of these mutations are discussed.

Due to the limited availability of experimental data, in simulations it was assumed that: the kinetic effect of the mutants on *I*_Kur_ is homogeneous throughout the atria; the mutants do not affect the relative response of *I*_Kur_ to beta stimulation; and, the mutant variants of *I*_Kur_ differ from the WT in the same way for both lone and chronic AF. Moreover, we assumed that these mutations do not directly induce structural remodelling including fibrosis/atrial enlargement. Such assumptions require further experimental verifications.

There are multiple confounding factors associated with the complex excitation patterns observed during AF which were not included in the present study in order to isolate the electrical effects of *I*_*Kur*_ modifications: (i) the model includes only idealised fibre structure along the main bundles of the CT/BB and PMs–previous studies have demonstrated a significant role of fibres in determining complex excitation patterns [15,36,37]; (ii) there is no inclusion of fibrosis or regional variation in diffusion coefficient–previous studies have demonstrated the important role of fibrosis and heterogeneous conduction velocity in the breakdown on excitation wavefronts [29,30]; (iii) atrial dilation and hypertrophy are not included and these may also promote complex excitation patterns.

Without inclusion of these factors, the complex and multi-wavelet excitation patterns underlying AF would not be expected to arise. However, the electrical mechanism underlying increased vulnerability to sustained arrhythmia is the same in both conditions: namely, shortening of the effective refractory period and consequently excitation wavelength, permitting faster and shorter re-entrant waves to be maintained. It is therefore reasonable to assume that promotion of re-entrant activity compared to control is a good indication for the isolated electrical modulation of vulnerability to complex excitation underlying AF. Moreover, once re-entrant excitation is initiated (i.e. a mother rotor) the additional factors described above will contribute to the breakdown of the excitation waves leading to the complex activity associated with AF.

Single cell models used in the present study are not suited for investigating delayed-after-depolarisations induced by spontaneous calcium release events, which are being increasingly linked to AF triggers [38]. Loss-of-function mutations were associated with increased incidence of EADs, and APD prolongation and EADs are both linked to increased SR loading and probability of spontaneous release events. These phenomena may play an important role in arrhythmia associated with loss-of-function mutations but could not be investigated in the present study.

The human SAN model implemented is not rigorously based on detailed electrophysiological data from human. However, the model was used in the present study to provide an accurate excitation driving force associated with sinus rhythm (i.e. rate and AP magnitude) and is deemed suitable for this purpose due to the faithful reproduction of SAN AP morphology and conduction. Tissue simulations for the SAN were only performed in 1D because extension to 3D requires the inclusion of additional factors determining SAN excitation patterns [39], beyond the scope of this study, which will have a large influence on the potential generation of unidirectional conduction patterns.

### Conclusion

Genetic variation in *KCNA5* promoted multiple mechanisms of arrhythmogenesis for both gain- and loss-of-function mutations, pertaining to the development of re-entrant excitation and early-after-depolarisations. Atrial AP morphology was demonstrated to be highly sensitive to *I*_Kur_ activation kinetics and had a strong impact on tissue dynamics.

## Methods

### Development of a novel formulation of *I*_Kur_

A new formulation of *I*_Kur_ was incorporated into the *Colman et al*. model based on experimental data from the wild-type (WT) K_V_1.5 channel [2]. The new formulation of *I*_Kur_ is as follows:

$$K_{Q10}=3.52$$ (1)

Activation gate:

$$a_{inf}=\frac{1.0}{1.0+\exp\left(\frac{V+17.67}{-5.75}\right)}\cdot \frac{1.0}{1.0+\exp\left(\frac{V+8.45}{-11.51}\right)}$$ (2)

$$\tau_{a}=\left(\frac{45.67}{1.0+\exp\left(\frac{V+11.23}{11.53}\right)}+4.27\right)\;\left(\frac{0.26}{1.0+\exp\left(\frac{V+35.87}{-3.88}\right)}+0.29\right)$$ (3)

$$\tau_{a}=\tau_{a}/K_{Q10}$$ (4)

$$\frac{da}{dt}=\frac{a_{\inf}-a}{\tau_{a}}$$ (5)

Inactivation gate:

$$i_{inf}=\frac{0.52}{1.0+\exp(\frac{V-15.11}{7.57})}+0.46$$ (6)

$$\tau_{i}=\frac{2328}{1.0+\exp(\frac{V-9.44}{3.58})}+1739.14$$ (7)

$$\tau_{i}=\tau_{i}/K_{Q10}$$ (8)

$$\frac{di}{dt}=\frac{i_{\inf}-i}{\tau_{i}}$$ (9)

*I*_Kur_ current:

$$I_{Kur}=0.64\left(4.51+\frac{1.90}{1.0+\exp\left(\frac{V-20.52}{-8.27}\right)}\right)a\cdot i\cdot (V-E_{K})$$ (10)

**Fig 4:**
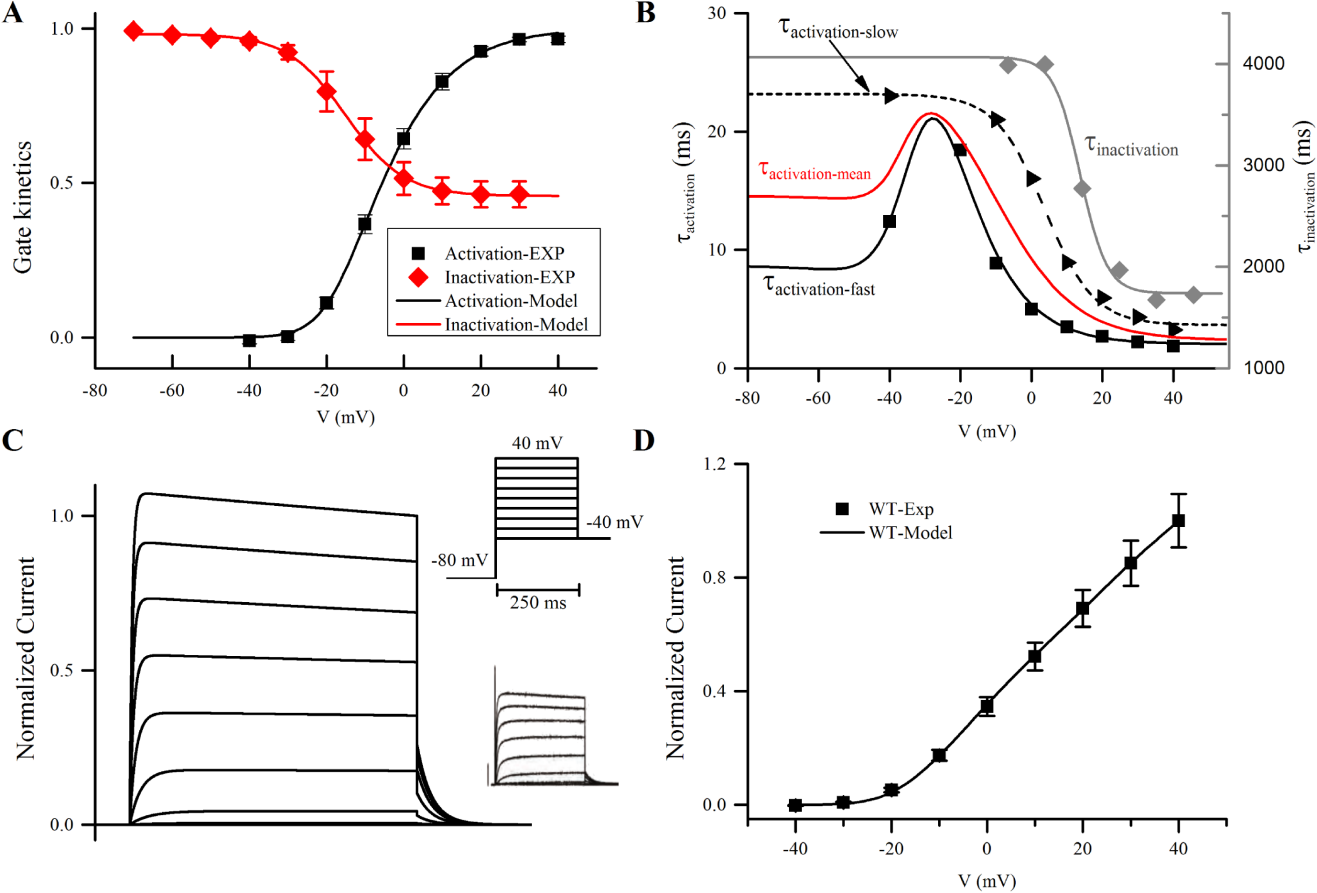
Model representation describing *I*_Kur_ and simulated voltage clamp. **A** Steady-state activation and inactivation; **B** Time constants; **C** Current trace obtained from a simulated voltage clamp. Inserts: top right–voltage protocol; bottom right–experimental current traces; **D** I-V relationship. In the Fig the simulation data were shown using lines, and experimental data represented by points. Experimental data were taken from [2]; specially, for **B** the time constants were derived from current traces from [2] by fitting the activation phase of the current trace to a mono-exponential equation.

The new model mediates an explicit dependence of channel conductivity to the membrane voltage. The maximum channel conductance was tuned so that the ratio of maximum current densities of *I*_to_ (the transient outward potassium current) and *I*_Kur_ is close to experimental observations [40]. In accordance with a previous study [21], Q10 correction was carried out to account for the difference in the temperature at which the experiments were carried out and physiological conditions. The new formulation of *I*_Kur_ closely reproduces the kinetics of the current in experimental conditions (Fig 4). Note that the experimental traces of voltage clamp for *I*_Kur_ show typically two types of activation–fast and slow (Fig 4B)–for different individual isolated single cells. In the new *I*_Kur_ model, the time constants of activation gate were modelled by the weighted mean of the fast and slow kinetics observed in the raw data set from the study [2], representing the homogenising effects of coupled cells in tissue.

**Fig 5:**
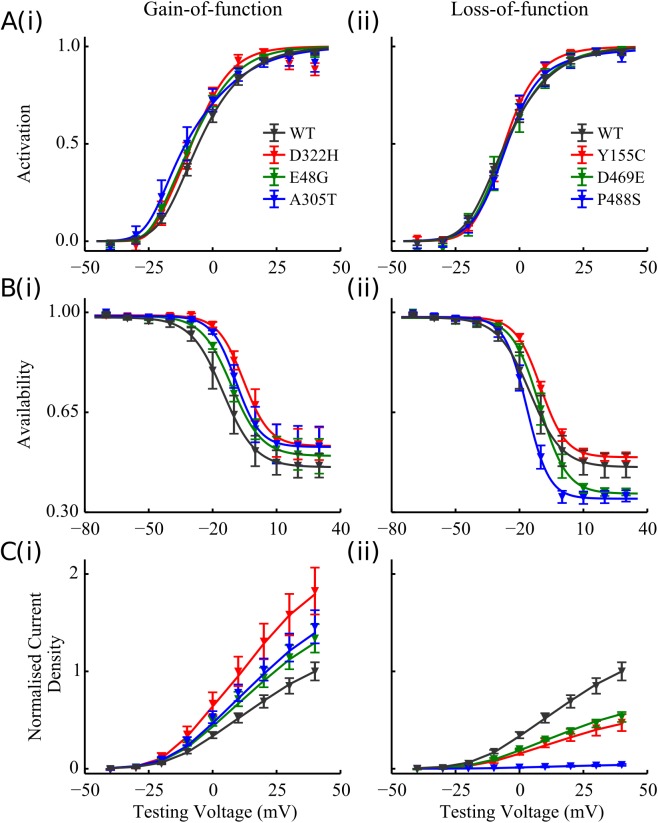
Modelling the electrophysiological properties of *I*_Kur_ carried by the mutants. **A** Steady-state activation; **B** Steady-state inactivation; **C** I-V relationship of *I*_Kur_ elicited by voltage clamp step protocol for the (i) gain- and (ii) loss-of-function mutations, compared to the WT (black). In the plots points indicate the experimental data [2] and lines represent simulated results.

To obtain models of the six *KCNA5*-mutant currents, parameters of channel conductance, activation and inactivation gates were fitted to individual mutation data from [2], whereas the time constants were kept the same with the WT model. The resultant activation, inactivation gates and I-V relationship measured from simulated voltage clamp are presented in Fig 5.

### Single cell models

Multiple contemporary models of human atrial electrophysiology (*Courtemanche et al*. [21]; *Grandi et al*. [17]; and *Colman et al*. [13]) were used in the present study. These models exhibit diverse human atrial action potential properties and profiles.

The novel formulation of *I*_Kur_ was incorporated into the *Colman et al*. model; the updated model *Colman et al*. model was validated against experimental data (S6 Text). In *Courtemanche et al*. and *Grandi et al*. models, effects of mutations on atrial electrophysiology were simulated by incorporating the relative changes in steady-state gates and conductance relative to the WT *I*_Kur_, summarised in Table 1.

**Table 1:** Changes in parameters of steady-state variables and maximum conductance of *I*_Kur_ carried by the *KCNA5* mutants relative to WT.

		D332H	E48G	A305T	Y155C	D469E	P488S
**Activation**	**V**_**1/2**_	-3.264	-3.024	-4.068	-0.893	0	0
	***K***	× 0.809	× 0.956	× 1.101	× 0.757	× 1	× 0.834
**Inactivation**	**V**_**1/2**_	+ 9.615	+ 4.028	+ 6.032	+ 5.013	+ 4.545	- 1.415
	***K***	× 0.801	× 0.940	× 0.748	× 0.782	× 0.842	× 0.688
	**Minimum Availability** **(*MA*)**	+ 0.074	+ 0.039	+ 0.071	+ 0.035	- 0.093	- 0.110
**Maximum Conductance**		× 1.799	× 1.323	× 1.445	× 0.475	× 0.546	× 0.038

Steady-state activation and inactivation were described using Bolzmann equation. For activation: *I*/*I*_*max*_ = 1/{1 + exp[(*V* − *V*_1/2_)/*k*]}, inactivation *I*/*I*_*max*_ = (1 − *MA*)/{1 + exp[(*V* − *V*_1/2_)/*k*]} + *MA*. Parameters were fitted to the experimental data digitalised from [2].

S1-S2 stimulation protocol was used to obtain the APD restitutions. For each model, a train of 100 S1 stimuli of 1000 ms was applied, which was followed by a further stimulus delayed by S2.

Regional cell models, accounting for variation in APD and AP morphology of difference regions within the atria, were implemented for the *Colman et al*. and *Courtemanche et al*. models from previous modelling studies [13,21] (S7 Text). Regional differences between the electrophysiology of the right atrial bundles of the crista terminalis (CT) and the pectinate muscles (PM) were also incorporated into the *Grandi et al*. model [17] based on the same data (S7 Text).

To study the impact of the mutations on EAD production, the effect of isoprotenerol (ISO, a β-agonist reproducing sympathetic activity) was incorporated into the *Grandi et al*. model according to their original study [17] and into the *Courtemanche et al*. and *Colman et al*. models based on the same parameter modifications.

Models of the *KCNA5* mutants were also combined with models of chronic AF-induced electrical remodelling (cAF) (from previous studies[13,17]), affecting the conductance and kinetics of multiple ion channels, in order to provide insight into the integral action of the arrhythmogenic substrate associated with the mutations and that associated with chronic AF (S8 Text).

### Tissue modelling

#### Mathematical model of electrical excitation wave propagation

The wave propagation of atrial excitation was modelled by the well-known reaction-diffusion equation [13], given by:

$$\frac{\partial V_{m}}{\partial t}=\nabla \cdot (D\;\nabla V_{m})-\frac{I_{ion}}{C_{m}}$$ (11)

where *V*_m_ is the membrane potential, ***D*** the tensor of diffusion coefficient, *I*_ion_ the total ionic transmembrane current in a single cell and the *C*_m_ is the membrane capacitance of the cell.

#### 1D strand models

A simplified tissue model is developed in which a string of single cells are coupled in a 1D strand. Half of the model is the CT and half the PM, representing this region of the right atrium. The diffusion coefficient is set to give a conduction velocity close to the experimentally observed values along the fibre direction of 1.3 m/s. The *Grandi et al*. model was implemented for the purpose of evaluating the behaviour of EADs in tissue.

We also constructed 1D strand models of the conduction pathways from the SAN to the surrounding atria. The CT/PM/RAA cell models are from the *Grandi et al*., as described previously (S7 Text). The SAN cell model was derived from the *Colman et al*. model and is an identical implementation to previous publications [14,41], based on mRNA data [42,43]. The morphology of simulated AP in the SAN model is comparable to experimental traces (Figure A in S9 Text).

#### 3D anatomical human atrial model

Our previously developed 3D virtual human atria [13,14,44] was applied to assess the functional impact of the mutations on electrical wave behaviour in the organ. Details of the model have been previously published [13,14] and are introduced in S10 Text. Briefly, the anatomical atrial model is derived from the visible human dataset [44] and segmented into the major differentiated anatomical regions [13,44]. For all simulations, the model was preconditioned by implementing the initial conditions for all state variables as the stable-state values from a 1D tissue strand paced at 1Hz for 100 beats. Sinus rhythm was simulated by pacing the atria from the sinoatrial node region. APD was measured to an accuracy of 1 ms at each node on the final of 10 beats at 1Hz, using the time at which voltage reaches above -40 mV as the upstroke time.

#### 3D Modelling of atrial vulnerability to unidirectional conduction block

**Fig 6:**
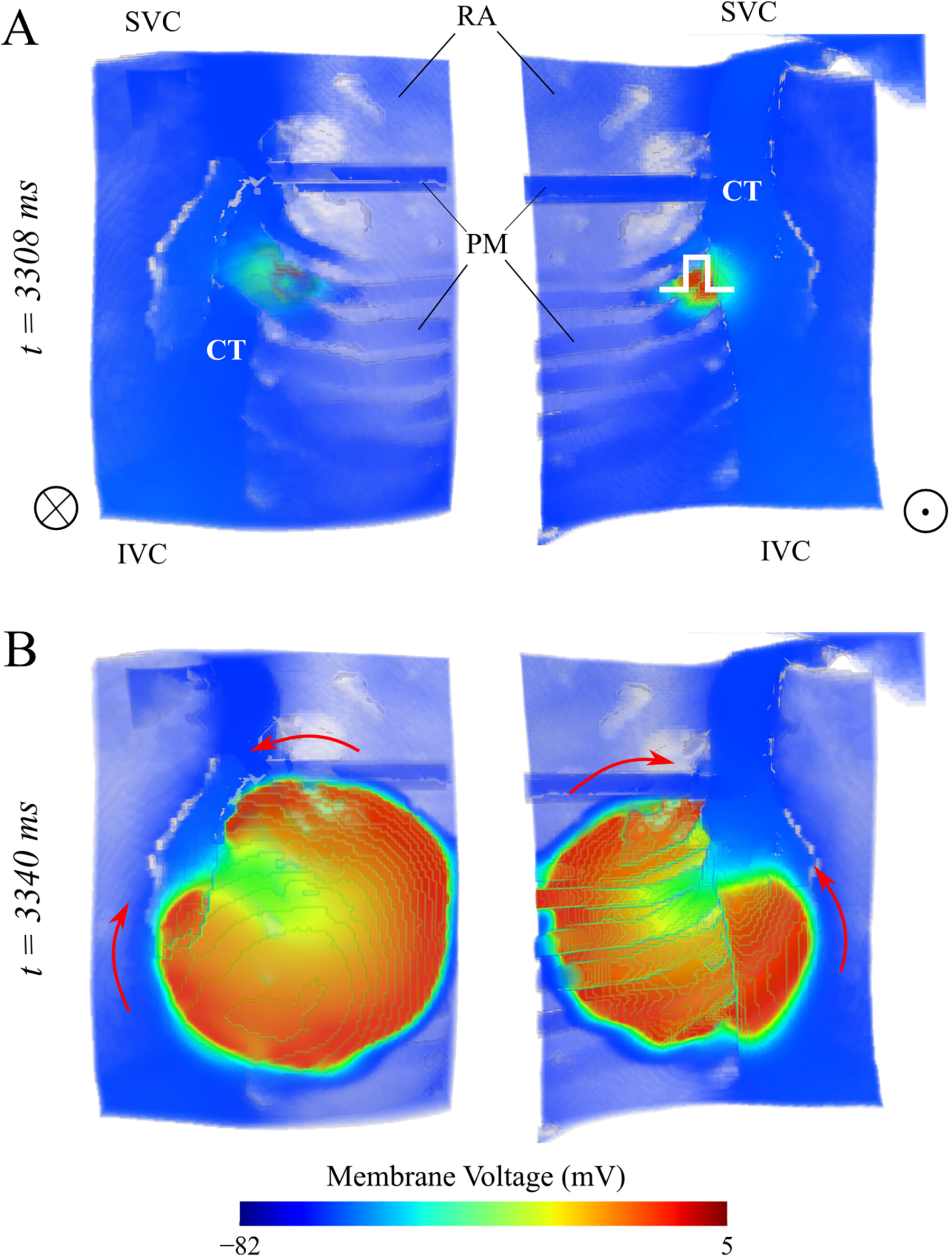
An example of simulated excitation wave break following S1-S2 stimuli at the CT/PM junction. **A** Volume rendering of the 3D human atrial wedge used in the simulations; S1-S2 were applied to the junction of CT/PM (indicated with white square wave in the top right panel); the labels are: SVC/IVC–superior/inferior vena cava, RA–right atrium, PM–pectinate muscle, CT–crista terminalis; **B** Simulated wave break arising from the S2 stimulus shown in **A**; the arrows indicate the direction of wave propagation.

Atrial vulnerability to unidirectional conduction block was quantified by an S1S2 pacing protocol applied to the heterogeneous junction [13,14,45] of the CT with the PM within a 3D atrial wedge (Fig 6A). A train of 7 S1 stimuli at 500ms was applied to a spherical tissue region of 3.3 mm in radius, which was followed by an S2 at variant intervals applied to the same site. This protocol has been used in previous studies [13]. The temporal vulnerability window was defined as the temporal range of S2 giving rise to wave break in either direction but not both. An example of simulated wave break is presented in Fig 6B.

#### Initiation of re-entry in the human atria

To study the effect of the mutation induced alterations to parameters of AP on re-entrant wave dynamics, a phase-distribution method was implemented in order to attain re-entrant excitation independent of initiation methods [46,47]. Whereas this initiation mechanism is artificial, it allows for the investigation of the long term dynamics of excitation.

**Fig 7:**
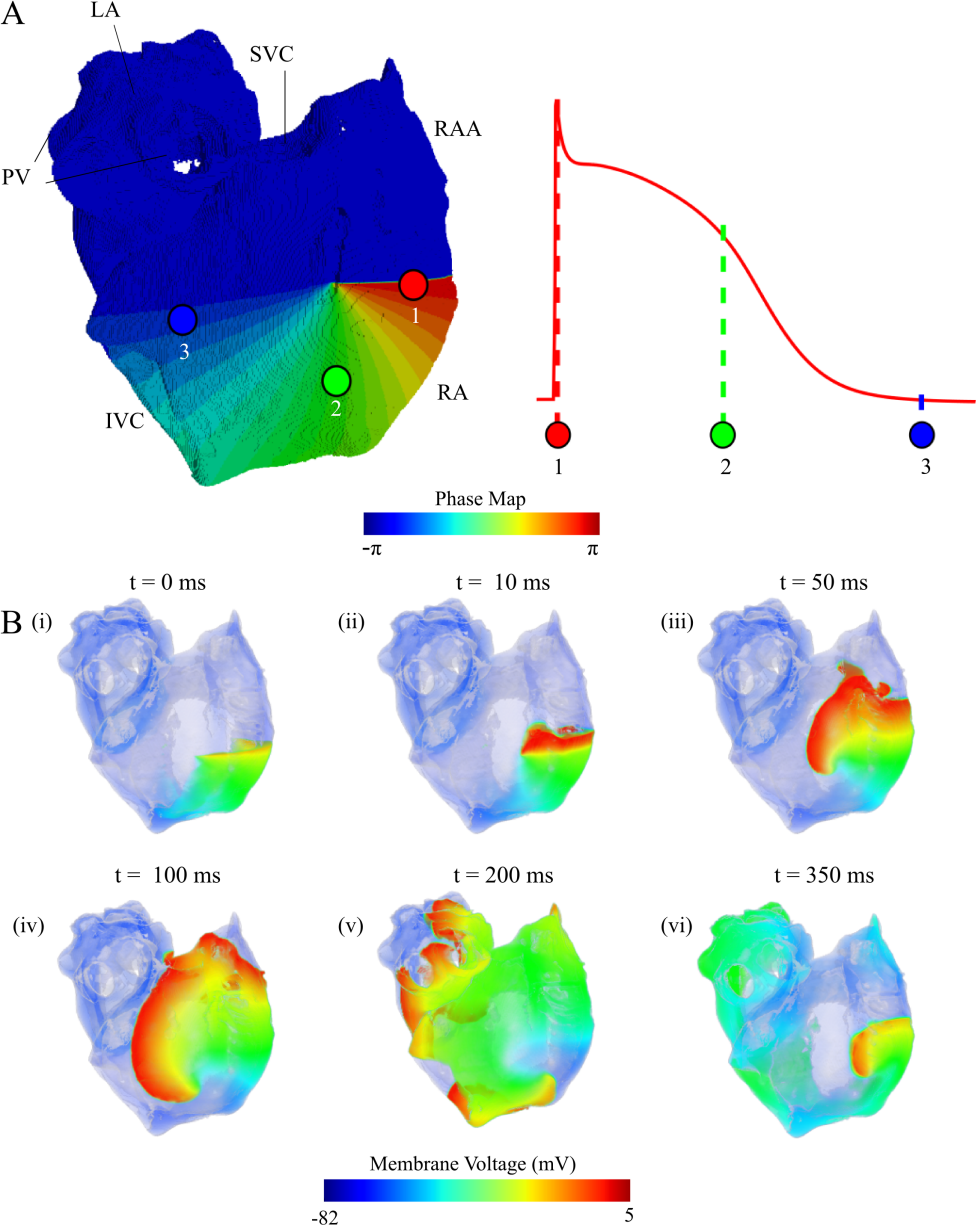
Illustration of the phase distribution method to initiate re-entrant waves in the 3D human atrial model. **A** The distributed phase map used on the anatomical model, with numbered indications of the stage of the AP which is mapped onto each location; RAA–right atrial appendage, PV–pulmonary vein, LA–left atrium; **B** Demonstration of the development of a spiral wave in the tissue with mapped initial conditions; the time of screenshot is indicated in the top of each panel.

The phase distribution method involves mapping the state variables from the single cell model onto the anatomical model to create an asymmetric conduction pattern: (1) export the state variables from the resultant AP of the final stimulus applied to a 1D strand of homogeneous tissue, at discrete intervals throughout an entire AP cycle (Fig 7A); (2) use these values as the initial conditions in the 3D model, spatially distributed throughout the anatomy to create an asymmetric propagation wave which will develop into a spiral/scroll (Fig 7B).

Note that in simulations the diffusion coefficient (***D***, diffusion tensor in the monodomain equation) in the model, mimicking the intercellular electrical coupling, was varied between 100% (control, lone AF) and 40% (severe remodelling) to simulate possible remodelling in the connexin and atrial structures (e.g. 30% increase in the atrial volume) associated with AF [48–50], which resulted in up to 40% reduction in the conduction velocity as seen in previous studies [49,51,52]. A reduction in ***D*** promotes sustained re-entry, and thus the extent of the reduction required to sustain re-entry is an indicator of vulnerability. The dominant frequencies (DF) of sustained re-entrant excitation were then computed through Fourier analysis of the electrical activity in multiple single cells in different regions throughout the tissue [13]. DF computation was used as a measure of the ability of the atria to sustain rapid excitation; detailed spatial DF maps to demonstrate regional variation in excitation frequency and identify driving rotors were not computed due to (i) the direct control over the initiation of re-entry and (ii) the single mother rotor excitation observed (see Results).

For each mutation and WT with *Colman et al*. and *Courtemanche et al*. models, the first 5000 ms following initiation of re-entry was simulated and recorded for analysis.

A description of the numerical methods used in this study is given in S11 Text.

## Supporting information

S1 Text Analysis of the role of *I*_Kur_ in human atrial AP morphology. (DOCX)

S2 Text Effects of chronic AF remodelling on single cell electrophysiology. (DOCX)

S3 Text Simulated re-entrant spiral wave in the 3D human atria. (DOCX)

S4 Text Vulnerability windows in the *Courtemanche et al*. model. (DOCX)

S5 Text Effects of beta-adrenergic stimulation on human atrial myocytes. (DOCX)

S6 Text Validations to the updated *Colman et al*. model with a new formulation of *I*_Kur._ (DOCX)

S7 Text Regional cell models. (DOCX)

S8 Text Modelling chronic-AF induced electrical remodelling. (DOCX)

S9 Text Human SAN model. (DOCX)

S10 Text 3D anatomical model of the human atria. (DOCX)

S11 Text Numerical methods. (DOCX)

## References

[1] Fatkin D, Seidman CE, Seidman JG. Genetics and Disease of Ventricular Muscle. Cold Spring Harb Perspect Med. 2014;4: a021063 doi: 10.1101/cshperspect.a021063 24384818 10.1101/cshperspect.a021063 PMC3869277

[2] Christophersen IE, Olesen MS, Liang B, Andersen MN, Larsen AP, Nielsen JB, et al Genetic variation in KCNA5: impact on the atrial-specific potassium current IKur in patients with lone atrial fibrillation. Eur Heart J. 2013;34: 1517–1525. doi: 10.1093/eurheartj/ehs442 23264583 10.1093/eurheartj/ehs442

[3] Andrade J, Khairy P, Dobrev D, Nattel S. The Clinical Profile and Pathophysiology of Atrial Fibrillation Relationships Among Clinical Features, Epidemiology, and Mechanisms. Circ Res. 2014;114: 1453–1468. doi: 10.1161/CIRCRESAHA.114.303211 24763464 10.1161/CIRCRESAHA.114.303211

[4] Eckstein J, Conen D, Kuehne M. Atrial fibrillation: A moving target. Swiss Med Wkly. 2014; doi: 10.4414/smw.2014.14078 25539064 10.4414/smw.2014.14078

[5] Nattel S. New ideas about atrial fibrillation 50 years on. Nature. 2002;415: 219–226. doi: 10.1038/415219a 11805846 10.1038/415219a

[6] Nattel S, Burstein B, Dobrev D. Atrial Remodeling and Atrial Fibrillation Mechanisms and Implications. Circ Arrhythm Electrophysiol. 2008;1: 62–73. doi: 10.1161/CIRCEP.107.754564 19808395 10.1161/CIRCEP.107.754564

[7] Workman AJ, Kane KA, Rankin AC. The contribution of ionic currents to changes in refractoriness of human atrial myocytes associated with chronic atrial fibrillation. Cardiovasc Res. 2001;52: 226–235. doi: 10.1016/S0008-6363(01)00380-7 11684070 10.1016/s0008-6363(01)00380-7

[8] Caballero R, de la Fuente MG, Gómez R, Barana A, Amorós I, Dolz-Gaitón P, et al In Humans, Chronic Atrial Fibrillation Decreases the Transient Outward Current and Ultrarapid Component of the Delayed Rectifier Current Differentially on Each Atria and Increases the Slow Component of the Delayed Rectifier Current in Both. J Am Coll Cardiol. 2010;55: 2346–2354. doi: 10.1016/j.jacc.2010.02.028 20488306 10.1016/j.jacc.2010.02.028

[9] Gonzalez de la Fuente M, Barana A, Gomez R, Amoros I, Dolz-Gaiton P, Sacristan S, et al Chronic atrial fibrillation up-regulates 1-Adrenoceptors affecting repolarizing currents and action potential duration. Cardiovasc Res. 2012;97: 379–388. doi: 10.1093/cvr/cvs313 23060133 10.1093/cvr/cvs313

[10] Schotten U, Haan S de, Verheule S, Harks EGA, Frechen D, Bodewig E, et al Blockade of atrial-specific K+-currents increases atrial but not ventricular contractility by enhancing reverse mode Na+/Ca2+-exchange. Cardiovasc Res. 2007;73: 37–47. doi: 10.1016/j.cardiores.2006.11.024 17157284 10.1016/j.cardiores.2006.11.024

[11] Pandit S V., Berenfeld O, Anumonwo JMB, Zaritski RM, Kneller J, Nattel S, et al Ionic Determinants of Functional Reentry in a 2-D Model of Human Atrial Cells During Simulated Chronic Atrial Fibrillation. Biophys J. 2005;88: 3806–3821. doi: 10.1529/biophysj.105.060459 15792974 10.1529/biophysj.105.060459 PMC1305615

[12] Kharche S, Garratt CJ, Boyett MR, Inada S, Holden A V., Hancox JC, et al Atrial proarrhythmia due to increased inward rectifier current (I(K1)) arising from KCNJ2 mutation—a simulation study. Prog Biophys Mol Biol. 2008;98: 186–197. doi: 10.1016/j.pbiomolbio.2008.10.010 19041665 10.1016/j.pbiomolbio.2008.10.010

[13] Colman MA, Aslanidi O V., Kharche S, Boyett MR, Garratt C, Hancox JC, et al Pro-arrhythmogenic effects of atrial fibrillation-induced electrical remodelling: insights from the three-dimensional virtual human atria. J Physiol. 2013;591: 4249–4272. doi: 10.1113/jphysiol.2013.254987 23732649 10.1113/jphysiol.2013.254987 PMC3779115

[14] Aslanidi O V., Colman MA, Stott J, Dobrzynski H, Boyett MR, Holden A V., et al 3D virtual human atria: A computational platform for studying clinical atrial fibrillation. Prog Biophys Mol Biol. 2011;107: 156–168. doi: 10.1016/j.pbiomolbio.2011.06.011 21762716 10.1016/j.pbiomolbio.2011.06.011 PMC3211061

[15] Colman MA, Varela M, Hancox JC, Zhang H, Aslanidi OV. Evolution and pharmacological modulation of the arrhythmogenic wave dynamics in canine pulmonary vein model. Europace. 2014;16: 416–423. doi: 10.1093/europace/eut349 24569896 10.1093/europace/eut349 PMC3934846

[16] Olson TM, Alekseev AE, Liu XK, Park S, Zingman L V., Bienengraeber M, et al Kv1.5 channelopathy due to KCNA5 loss-of-function mutation causes human atrial fibrillation. Hum Mol Genet. 2006;15: 2185–2191. doi: 10.1093/hmg/ddl143 16772329 10.1093/hmg/ddl143

[17] Grandi E, Pandit S V., Voigt N, Workman AJ, Dobrev D, Jalife J, et al Human Atrial Action Potential and Ca2+ Model Sinus Rhythm and Chronic Atrial Fibrillation. Circ Res. 2011;109: 1055–1066. doi: 10.1161/CIRCRESAHA.111.253955 21921263 10.1161/CIRCRESAHA.111.253955 PMC3208665

[18] Xie Y, Sato D, Garfinkel A, Qu Z, Weiss JN. So Little Source, So Much Sink: Requirements for Afterdepolarizations to Propagate in Tissue. Biophys J. 2010;99: 1408–1415. doi: 10.1016/j.bpj.2010.06.042 20816052 10.1016/j.bpj.2010.06.042 PMC2931729

[19] Qu Z, Xie L-H, Olcese R, Karagueuzian HS, Chen P-S, Garfinkel A, et al Early afterdepolarizations in cardiac myocytes: beyond reduced repolarization reserve. Cardiovasc Res. 2013;99: 6–15. doi: 10.1093/cvr/cvt104 23619423 10.1093/cvr/cvt104 PMC3687754

[20] Song Z, Ko CY, Nivala M, Weiss JN, Qu Z. Calcium-voltage coupling in the genesis of early and delayed afterdepolarizations in cardiac myocytes. Biophys J. 2015;108: 1908–1921. doi: 10.1016/j.bpj.2015.03.011 25902431 10.1016/j.bpj.2015.03.011 PMC4407256

[21] Courtemanche M, Ramirez RJ, Nattel S. Ionic mechanisms underlying human atrial action potential properties: insights from a mathematical model. Am J Physiol—Heart Circ Physiol. 1998;275: H301–H321. 10.1152/ajpheart.1998.275.1.H301 9688927

[22] Wettwer E, Hála O, Christ T, Heubach JF, Dobrev D, Knaut M, et al Role of IKur in Controlling Action Potential Shape and Contractility in the Human Atrium Influence of Chronic Atrial Fibrillation. Circulation. 2004;110: 2299–2306. doi: 10.1161/01.CIR.0000145155.60288.71 15477405 10.1161/01.CIR.0000145155.60288.71

[23] Almquist J, Wallman M, Jacobson I, Jirstrand M. Modeling the Effect of Kv1.5 Block on the Canine Action Potential. Biophys J. 2010;99: 2726–2736. doi: 10.1016/j.bpj.2010.08.062 21044569 10.1016/j.bpj.2010.08.062 PMC2965949

[24] Scholz EP, Carrillo-Bustamante P, Fischer F, Wilhelms M, Zitron E, Dössel O, et al Rotor Termination Is Critically Dependent on Kinetic Properties of IKur Inhibitors in an In Silico Model of Chronic Atrial Fibrillation. PLoS ONE. 2013;8: e83179 doi: 10.1371/journal.pone.0083179 24376659 10.1371/journal.pone.0083179 PMC3869770

[25] Tsujimae K, Murakami S, Kurachi Y. In silico study on the effects of IKur block kinetics on prolongation of human action potential after atrial fibrillation-induced electrical remodeling. Am J Physiol—Heart Circ Physiol. 2008;294: H793–H800. doi: 10.1152/ajpheart.01229.2007 18055524 10.1152/ajpheart.01229.2007

[26] Burashnikov A, Antzelevitch C. Can inhibition of IKur promote atrial fibrillation? Heart Rhythm. 2008;5: 1304–1309. doi: 10.1016/j.hrthm.2008.05.020 18774108 10.1016/j.hrthm.2008.05.020 PMC2632605

[27] Christ T, Wettwer E, Voigt N, Hála O, Radicke S, Matschke K, et al Pathology-specific effects of the IKur/Ito/IK,ACh blocker AVE0118 on ion channels in human chronic atrial fibrillation. Br J Pharmacol. 2008;154: 1619–1630. doi: 10.1038/bjp.2008.209 18536759 10.1038/bjp.2008.209 PMC2518460

[28] Tamargo J, Caballero R, Gómez R, Delpón E. IKur/Kv1.5 channel blockers for the treatment of atrial fibrillation. Expert Opin Investig Drugs. 2009;18: 399–416. doi: 10.1517/13543780902762850 19335273 10.1517/13543780902762850

[29] Morgan R, Colman MA, Chubb H, Seemann G, Aslanidi OV. Slow Conduction in the Border Zones of Patchy Fibrosis Stabilizes the Drivers for Atrial Fibrillation: Insights from Multi-Scale Human Atrial Modeling. Front Physiol. 2016;7 doi: 10.3389/fphys.2016.00474 27826248 10.3389/fphys.2016.00474 PMC5079097

[30] McDowell KS, Vadakkumpadan F, Blake R, Blauer J, Plank G, Macleod RS, et al Mechanistic inquiry into the role of tissue remodeling in fibrotic lesions in human atrial fibrillation. Biophys J. 2013;104: 2764–2773. doi: 10.1016/j.bpj.2013.05.025 23790385 10.1016/j.bpj.2013.05.025 PMC3686346

[31] Chang MG, Sato D, de Lange E, Lee J-H, Karagueuzian HS, Garfinkel A, et al Bi-stable wave propagation and early afterdepolarization–mediated cardiac arrhythmias. Heart Rhythm. 2012;9: 115–122. doi: 10.1016/j.hrthm.2011.08.014 21855520 10.1016/j.hrthm.2011.08.014 PMC3246094

[32] Wilhelms M, Maleckar MM, Koivumäki JT, Dössel O, Seemann G. Benchmarking electrophysiological models of human atrial myocytes. Front Comput Physiol Med. 2013;3: 487 doi: 10.3389/fphys.2012.00487 23316167 10.3389/fphys.2012.00487 PMC3539682

[33] Wang Z, Fermini B, Nattel S. Delayed rectifier outward current and repolarization in human atrial myocytes. Circ Res. 1993;73: 276–285. doi: 10.1161/01.RES.73.2.276 8330373 10.1161/01.res.73.2.276

[34] Nygren A, Fiset C, Firek L, Clark JW, Lindblad DS, Clark RB, et al Mathematical Model of an Adult Human Atrial Cell The Role of K+ Currents in Repolarization. Circ Res. 1998;82: 63–81. doi: 10.1161/01.RES.82.1.63 9440706 10.1161/01.res.82.1.63

[35] Voigt N, Heijman J, Wang Q, Chiang DY, Li N, Karck M, et al Cellular and Molecular Mechanisms of Atrial Arrhythmogenesis in Patients With Paroxysmal Atrial Fibrillation. Circulation. 2014;129: 145–156. doi: 10.1161/CIRCULATIONAHA.113.006641 24249718 10.1161/CIRCULATIONAHA.113.006641 PMC4342412

[36] Varela M, Colman MA, Hancox JC, Aslanidi OV. Atrial Heterogeneity Generates Re-entrant Substrate during Atrial Fibrillation and Anti-arrhythmic Drug Action: Mechanistic Insights from Canine Atrial Models. PLOS Comput Biol. 2016;12: e1005245 doi: 10.1371/journal.pcbi.1005245 27984585 10.1371/journal.pcbi.1005245 PMC5161306

[37] Butters TD, Aslanidi OV, Zhao J, Smaill B, Zhang H. A novel computational sheep atria model for the study of atrial fibrillation. Interface Focus. 2013;3: 20120067 doi: 10.1098/rsfs.2012.0067 24427521 10.1098/rsfs.2012.0067 PMC3638473

[38] Nattel S, Dobrev D. The multidimensional role of calcium in atrial fibrillation pathophysiology: mechanistic insights and therapeutic opportunities. Eur Heart J. 2012;33: 1870–1877. doi: 10.1093/eurheartj/ehs079 22507975 10.1093/eurheartj/ehs079

[39] Fedorov VV, Glukhov AV, Chang R, Kostecki G, Aferol H, Hucker WJ, et al Optical Mapping of the Isolated Coronary-Perfused Human Sinus Node. J Am Coll Cardiol. 2010;56: 1386–1394. doi: 10.1016/j.jacc.2010.03.098 20946995 10.1016/j.jacc.2010.03.098 PMC3008584

[40] Bosch RF, Zeng X, Grammer JB, Popovic K, Mewis C, Kühlkamp V. Ionic mechanisms of electrical remodeling in human atrial fibrillation. Cardiovasc Res. 1999;44: 121–131. doi: 10.1016/S0008-6363(99)00178-9 10615396 10.1016/s0008-6363(99)00178-9

[41] Colman MA. Development of a Family of Regional Cell Models. Mechanisms of Atrial Arrhythmias. Springer International Publishing; 2014 pp. 87–114. Available: http://link.springer.com/chapter/10.1007/978-3-319-01643-6_4

[42] Chandler NJ, Greener ID, Tellez JO, Inada S, Musa H, Molenaar P, et al Molecular architecture of the human sinus node: insights into the function of the cardiac pacemaker. Circulation. 2009;119: 1562–1575. doi: 10.1161/CIRCULATIONAHA.108.804369 19289639 10.1161/CIRCULATIONAHA.108.804369

[43] Chandler N, Aslanidi O, Buckley D, Inada S, Birchall S, Atkinson A, et al Computer three-dimensional anatomical reconstruction of the human sinus node and a novel paranodal area. Anat Rec Hoboken NJ 2007. 2011;294: 970–979. doi: 10.1002/ar.21379 21538926 10.1002/ar.21379

[44] Seemann G, Höper C, Sachse FB, Dössel O, Holden AV, Zhang H. Heterogeneous three-dimensional anatomical and electrophysiological model of human atria. Philos Transact A Math Phys Eng Sci. 2006;364: 1465–1481. doi: 10.1098/rsta.2006.1781 16766355 10.1098/rsta.2006.1781

[45] Aslanidi OV, Colman MA, Varela M, Zhao J, Smaill BH, Hancox JC, et al Heterogeneous and anisotropic integrative model of pulmonary veins: computational study of arrhythmogenic substrate for atrial fibrillation. Interface Focus. 2013;3: 20120069 doi: 10.1098/rsfs.2012.0069 24427522 10.1098/rsfs.2012.0069 PMC3638474

[46] Biktashev VN, Holden AV. Reentrant waves and their elimination in a model of mammalian ventricular tissue. Chaos Woodbury N. 1998;8: 48–56. doi: 10.1063/1.166307 12779709 10.1063/1.166307

[47] Kharche SR, Stary T, Colman MA, Biktasheva IV, Workman AJ, Rankin AC, et al Effects of human atrial ionic remodelling by β-blocker therapy on mechanisms of AF: a computer simulation. Eur Eur Pacing Arrhythm Card Electrophysiol J Work Groups Card Pacing Arrhythm Card Cell Electrophysiol Eur Soc Cardiol. 2014;16: 1524–1533. doi: 10.1093/europace/euu084 25085203 10.1093/europace/euu084 PMC4640177

[48] Severs NJ, Bruce AF, Dupont E, Rothery S. Remodelling of gap junctions and connexin expression in diseased myocardium. Cardiovasc Res. 2008;80: 9–19. doi: 10.1093/cvr/cvn133 18519446 10.1093/cvr/cvn133 PMC2533424

[49] Stiles MK, John B, Wong CX, Kuklik P, Brooks AG, Lau DH, et al Paroxysmal Lone Atrial Fibrillation Is Associated With an Abnormal Atrial Substrate: Characterizing the “Second Factor.” J Am Coll Cardiol. 2009;53: 1182–1191. doi: 10.1016/j.jacc.2008.11.054 19341858 10.1016/j.jacc.2008.11.054

[50] Bikou O, Thomas D, Trappe K, Lugenbiel P, Kelemen K, Koch M, et al Connexin 43 gene therapy prevents persistent atrial fibrillation in a porcine model. Cardiovasc Res. 2011;92: 218–225. doi: 10.1093/cvr/cvr209 21799069 10.1093/cvr/cvr209

[51] Igarashi T, Finet JE, Takeuchi A, Fujino Y, Strom M, Greener ID, et al Connexin Gene Transfer Preserves Conduction Velocity and Prevents Atrial Fibrillation. Circulation. 2012;125: 216–225. doi: 10.1161/CIRCULATIONAHA.111.053272 22158756 10.1161/CIRCULATIONAHA.111.053272 PMC3260348

[52] Platonov PG, Mitrofanova LB, Orshanskaya V, Ho SY. Structural Abnormalities in Atrial Walls Are Associated With Presence and Persistency of Atrial Fibrillation But Not With Age. J Am Coll Cardiol. 2011;58: 2225–2232. doi: 10.1016/j.jacc.2011.05.061 22078429 10.1016/j.jacc.2011.05.061

