# Supplementary material for: In silico assessment of genetic variation in KCNA5 reveals multiple mechanisms of human atrial arrhythmogenesis
Source: PLoS Comput Biol. 2017 Jun 16;13(6):e1005587. doi: 10.1371/journal.pcbi.1005587 (PMC5493429; doi:10.1371/journal.pcbi.1005587)
Supplement: S1 Text — (DOCX) [file pcbi.1005587.s001.docx]

# Supporting Information 1: Analysis of the role of *I*_Kur_ in human atrial AP morphology

## Description of the gain-of-function mutations

In the Lone AF condition (no electrical remodelling) the mutations A305T and E48G resulted in APD_90_ shortening in all models (Figure 1, MS) as well as a small loss of rate-adaption (Figure 2, MS). APD_90_ shortening is primarily a result of a decreased and less prominent plateau due to the increased activity of *I*_Kur_ during phase-2 (Figure A).

The mutation D322H resulted in heterogeneous effects on APD_90_ in different models: In the *Courtemanche* *et al.* model D322H results in the most significant APD shortening compared to the other gain-of-function mutations, A305T and E48G, and the WT; in the updated *Colman et al. model* the mutation results in a faster phase-1 repolarisation, a more negative and prolonged plateau and APD_90_ prolongation compared to the WT (Figure 2, MS); In the *Grandi* *et al.* model, D322H also results in more negative notch and plateau potentials, the APD_90_ is shortened compared to the WT (Figure 2, MS) but still longer than A305T, despite the increase in current density being greater.

APD_90_ prolongation in D322H in the *Colman* *et al.* model results from a combination of the fast deactivation of *I*_Kur_ (rapidly turning off the repolarising current at potentials below -40mV), and the more negative notch and plateau potentials (leading to incomplete activation of the terminal repolarising currents *I_Ks_* and *I_Kr_*) (Figure B). In the *Grandi et al.* model, D322H also results in more negative notch and plateau potentials, but the APD_90_ is shortened compared to the WT (Figure 2, MS). However, through the same mechanism which results in prolongation in the *Colman et al. model*, the APD shortening is not as severe as the mutation A305T, despite D322H resulting in a greater increase in the maximum current density.

Results in APD_30_ and plateau potential demonstrated a higher consistency among the three models (Figure A). All gain-of-function mutations resulted in reduced APD_30_ in the three models, and the updated Colman *et al.* model showed most significant effects on APD_30_ due to increased *I*_Kur_ current density. These gain-of-function mutations also led to more negative plateau potential, independent of whether APD_90_ was lengthened or shortened.

## Description of the loss-of-function mutations

In the *Colman* *et al.* and *Courtemanche* *et al.* models, the loss-of-function mutations result in a more prominent and humped dome at the plateau phase (Figure 1, MS). The secondary effect on the activation of *I_Ks_* and *I_Kr_* plays a key role the rate-dependent APD changes. P488S, in which *I*_Kur_ is most severely reduced, results in the most significant elevation of the plateau compared to the other mutations, Y155C and D469E. In the *Grandi* *et al.* model, type 3 triangular morphologies are observed for all mutant types, although the plateau is elevated and APD_90_ is prolonged. The mutation P488S also results in minor EAD-like oscillations during phase 2 (Figure 1, MS). Abbreviation or prolongation of APD_90_ varied between the models (Figure A). These results are consistent with previous experimental studies showing blocking *I*_Kur_ could either lead to an increase [1] or decrease [2] in APD of human atrial cells depending on the baseline morphology of the atrial AP.

APD_30_ was increased in all three models in the loss-of-function mutants. Also, these mutants shifted the plateau potential to more positive values in the all three models (Figure A). These results indicate positive link between plateau potential, which is consistent with the previous experimental study on blocking *I*_Kur_ [3].

**
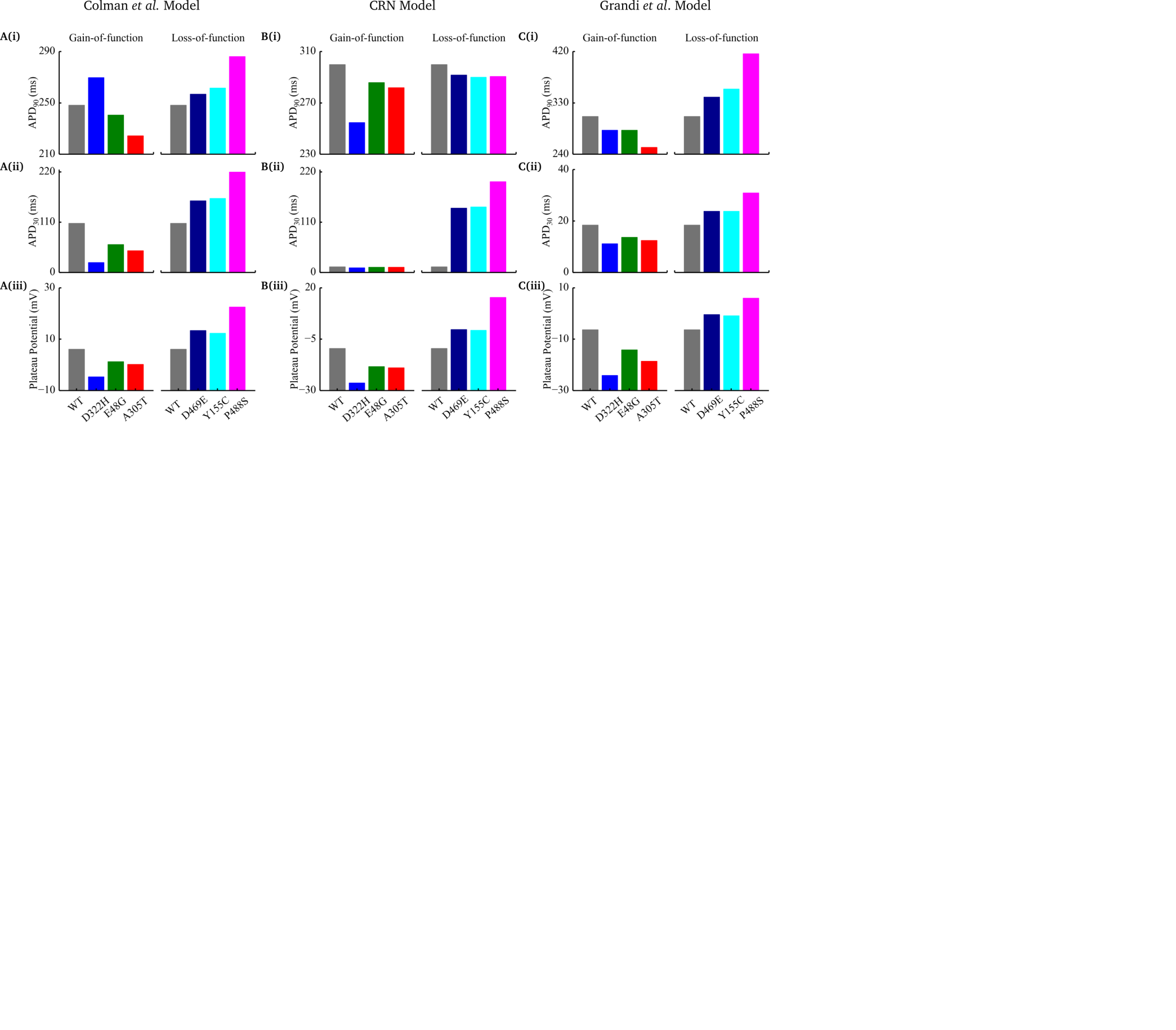
**

**Figure A** A summary of the effects of KCNA5 mutations on APD_90_, APD_30_ and plateau potential using the three human atrial electrophysiological models. Plateau potential was measured as the average value of potential within the window of 10 to 50 ms following the upstroke of action potentials [3].


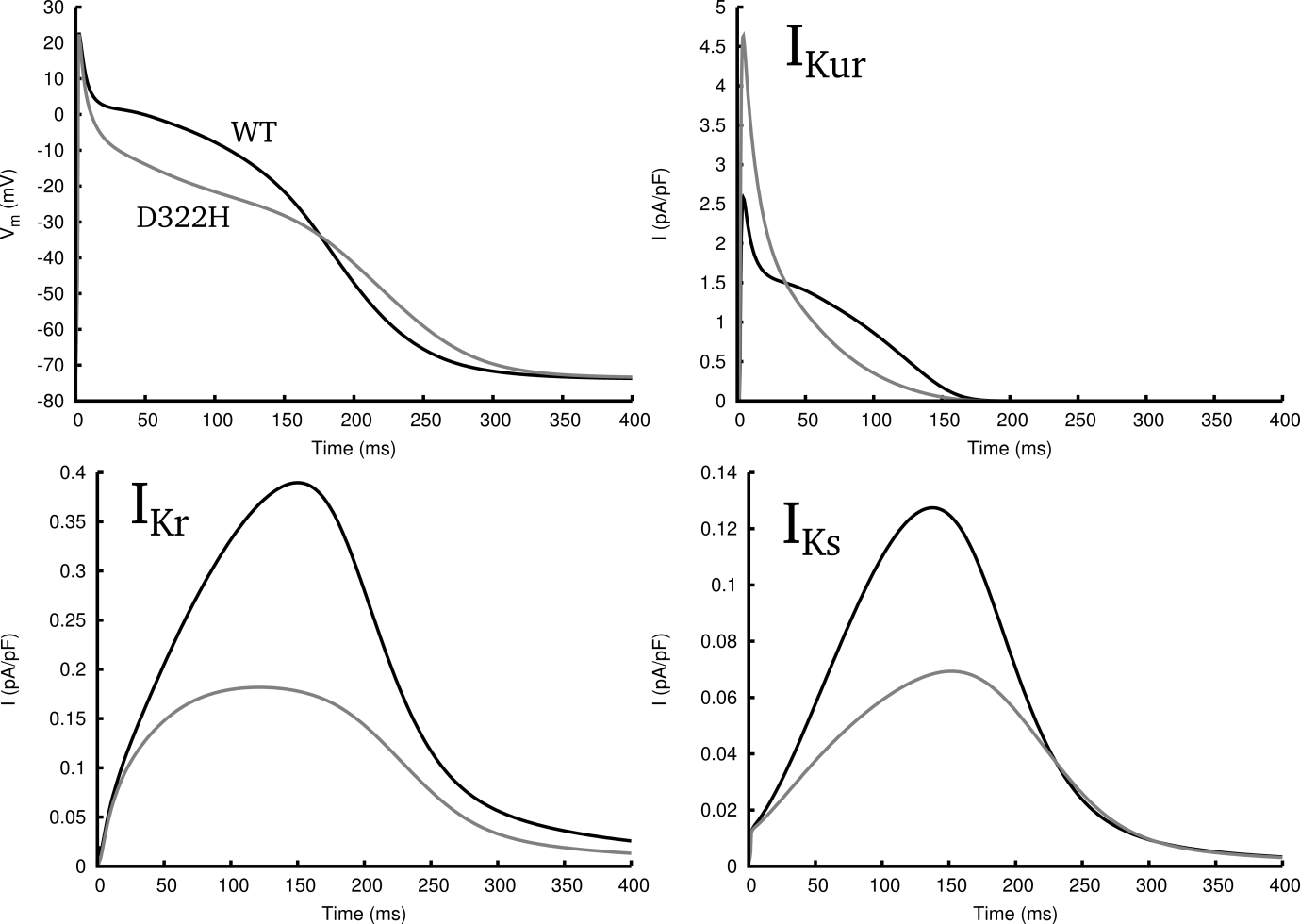


**Figure B** Potassium currents in D322H compared to WT in the *Colman* *et al.* model.

## Conductance and kinetics effects are heterogeneous

The relative contributions of the modifications of conductance and kinetics to the changes in AP morphology and duration were analysed by comparing the effects of each change individually with the WT and full mutation conditions.

In the gain-of-function mutations, modifications to the conductance alone resulted in a lower notch potential and APD prolongation in all cases, due to less activation of *I_Kr_* and *I_Ks_* (Figure C). Including the kinetic changes alone resulted in only a small change to the notch potential and shortening of the APD in all mutations, with D322H demonstrating the smallest change compared to A305T and E48G. This shortening is a result of the left-ward shift of the steady-state of activation curve, resulting in a higher open probability and therefore greater activity of *I*_Kur_ during phase 2 and 3, combined with the lack of effect on *I_Kr_* and *I_Ks_* due to only a small change in the plateau potential. D322H exhibited the smallest shift and therefore had the smallest effect. Hence, in terms of APD_90_, conductance and kinetics had opposing effects; D322H showed the largest conductance and smallest kinetic effect, explaining why in this mutation prolongation is observed whereas shortening is observed for the other two (in the *Colman* *et al.* model).

In the loss-of-function mutations D469E and P488S, changes to the activation kinetics are insignificant (note that inactivation of *I*_Kur_ plays only a small role and hence changes to inactivation kinetics are not important) and hence inclusion of these changes alone has no significant effect on the AP; AP effects are dominated by the decrease of conductance. However, Y155C demonstrates heterogeneous properties; the activation curve is shifted slightly to the left at potentials above -10 mV, and slightly to the right at potential below -10 mV. This results in a slight lowering of the notch and prolongation of the AP due to *I*_Kur_ turning off.

## General Analysis of the dependence of APD on *I*_Kur_ kinetics

We further analyse the relationship between the time-constant, the conductance of *I*_Kur_ and the resulting change in APD_90_ relative to the WT values with different V_1/2_ of *I*_Kur_ activation using the *Colman et al. model* (Figure D). It was demonstrated that increased time-constant lead to shortened APD_90,_ whereas the conductance rendered more complex behaviour. For models with positively shifted V_1/2_ of *I*_Kur_ kinetics, APD_90_ was extended with either increased or decreased channel conductance of *I_Kur_*. Similar effects were observed with negatively shifted V_1/2_ of *I*_Kur_ kinetics and smaller time-constant. Furthermore, the impact of variations in conductance and time-constants of activation was enhanced with negatively shifted V_1/2_.

These variations result from the complex interplay of multiple factors mitigating AP morphology: increased/decreased *I_Kur_* always increased/decreased the AP plateau, which decreased/increased the activity of *I_Ks_* and *I_kr_*. The extent of these secondary effects is determined by the specific AP morphology and the voltage dependence of *I_Ks_* and *I_kr_.* Finally, in the case for increased *I_Kur_* and associated decreased *I_Ks_* and *I_Kr_*, the time constant of *I_Kur_* activation determines its deactivation rate: if rapid, then there is a significant loss of repolarising current in the final phase of the AP, extending the tail and prolonging APD_90_.


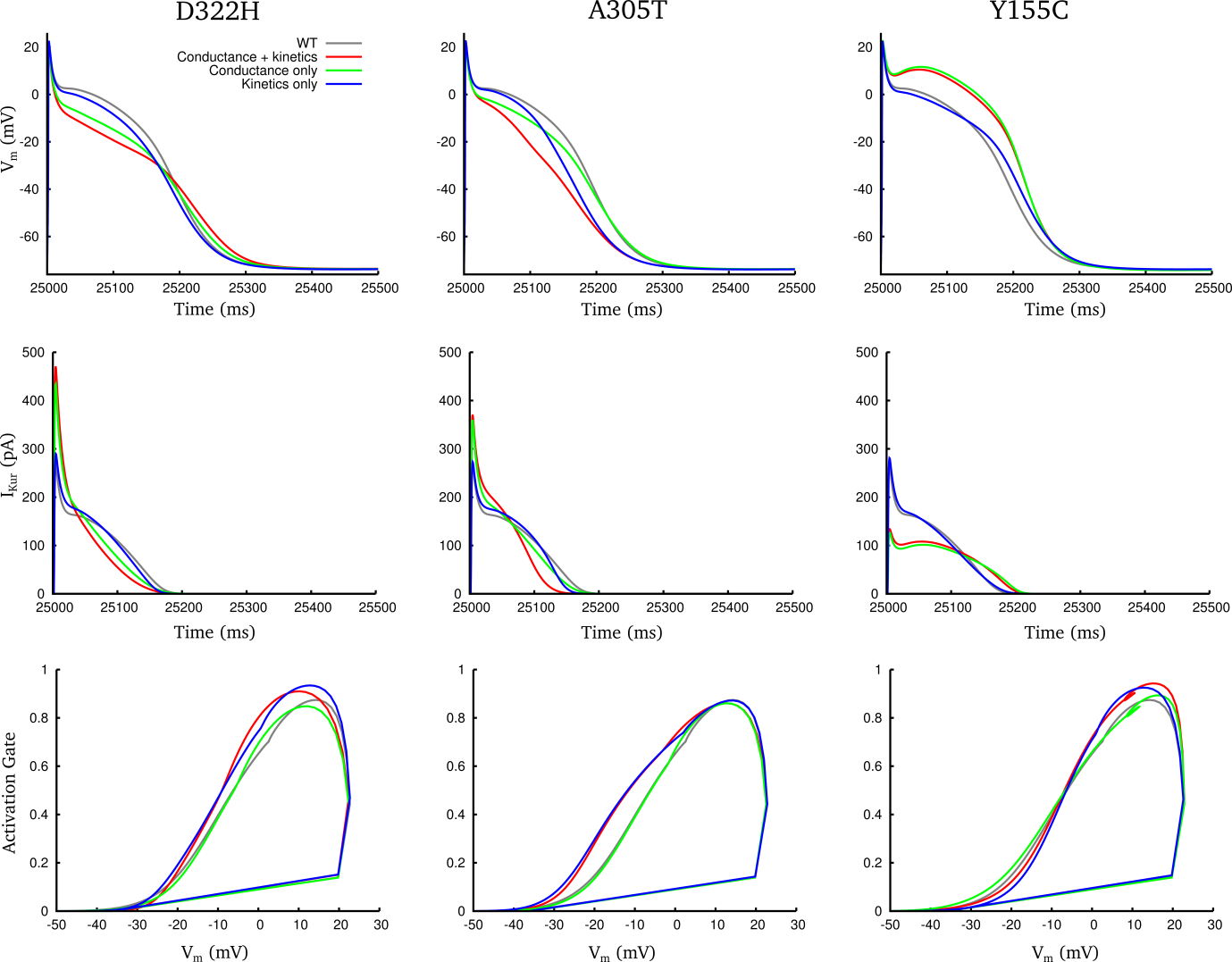


**Figure C** Conductance vs kinetics effects for three mutations in the *Colman et al. model*.


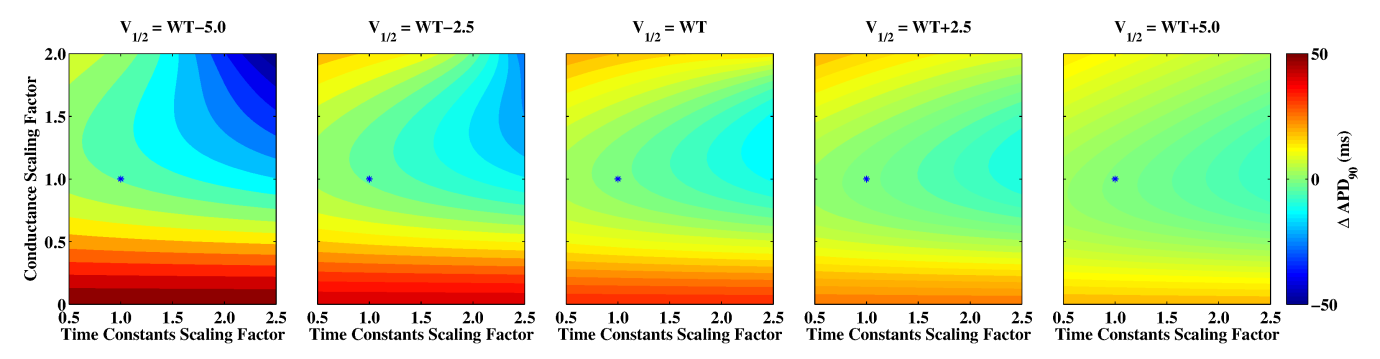


**Figure D** Analysis of the relationship between APD_90_ and the *I*_Kur_ channel abundance, time constant and V_1/2_ of *I*_Kur_ activation kinetics. Simulations were implemented using Colman *et al.* model.

## References

1. Workman AJ, Kane KA, Rankin AC. The contribution of ionic currents to changes in refractoriness of human atrial myocytes associated with chronic atrial fibrillation. Cardiovasc Res. 2001;52: 226–235. doi:10.1016/S0008-6363(01)00380-7

2. Wettwer E, Hála O, Christ T, Heubach JF, Dobrev D, Knaut M, *et al.* Role of IKur in Controlling Action Potential Shape and Contractility in the Human Atrium Influence of Chronic Atrial Fibrillation. Circulation. 2004;110: 2299–2306. doi:10.1161/01.CIR.0000145155.60288.71

3. Schotten U, Haan S de, Verheule S, Harks EGA, Frechen D, Bodewig E, *et al.* Blockade of atrial-specific K+-currents increases atrial but not ventricular contractility by enhancing reverse mode Na+/Ca2+-exchange. Cardiovasc Res. 2007;73: 37–47. doi:10.1016/j.cardiores.2006.11.024
