# Supplementary material for: In silico assessment of genetic variation in KCNA5 reveals multiple mechanisms of human atrial arrhythmogenesis
Source: PLoS Comput Biol. 2017 Jun 16;13(6):e1005587. doi: 10.1371/journal.pcbi.1005587 (PMC5493429; doi:10.1371/journal.pcbi.1005587)
Supplement: S2 Text — (DOCX) [file pcbi.1005587.s002.docx]

# Supporting Information 2: Effects of chronic AF remodelling on single cell electrophysiology

The functional impact of the mutations on single-cell AP morphology in chronic AF (cAF) conditions was consistent between groups of mutations and cell models: gain-of-function mutations shortened the APD whereas loss-of-function mutations prolonged the APD compared to the remodelled WT (Figure A).


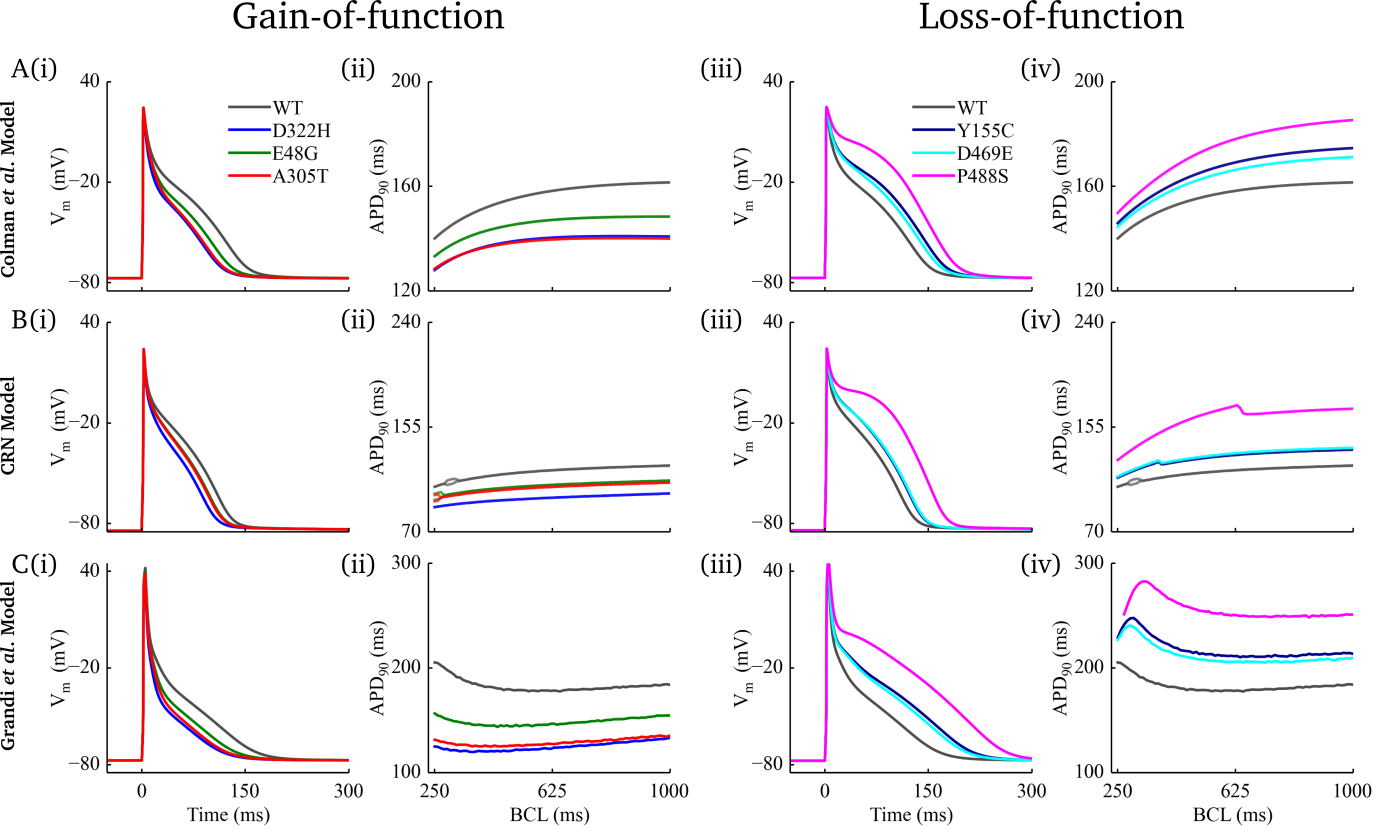


**Figure A.** Effects of *KCNA5* mutations on AP and APD restitution in the presence of cAF remodelling in the three cell models. The three rows show results obtained using (A) Colman *et al.*, (B) Courtemanche *et al.* (CRN) and (C) Grandi *et al.* models.
