# Supplementary material for: In silico assessment of genetic variation in KCNA5 reveals multiple mechanisms of human atrial arrhythmogenesis
Source: PLoS Comput Biol. 2017 Jun 16;13(6):e1005587. doi: 10.1371/journal.pcbi.1005587 (PMC5493429; doi:10.1371/journal.pcbi.1005587)
Supplement: S3 Text — (DOCX) [file pcbi.1005587.s003.docx]

# Supporting Information 3: Simulated re-entrant spiral wave in the 3D human atria

Figure A shows two snapshots of simulated re-entry using *Colman et al.* model. Using the same model, the mutation D322H increased APD_90_ at slow pacing rates (MS Figure 1A) while accelerating the frequencies of re-entrant excitation waves (MS Figure 1Bi). A further investigation into the time courses of AP generated by right atrial myocytes during the simulated re-entry revealed that the APD with D322H was shortened, while the excitation rate was increased compared with WT.


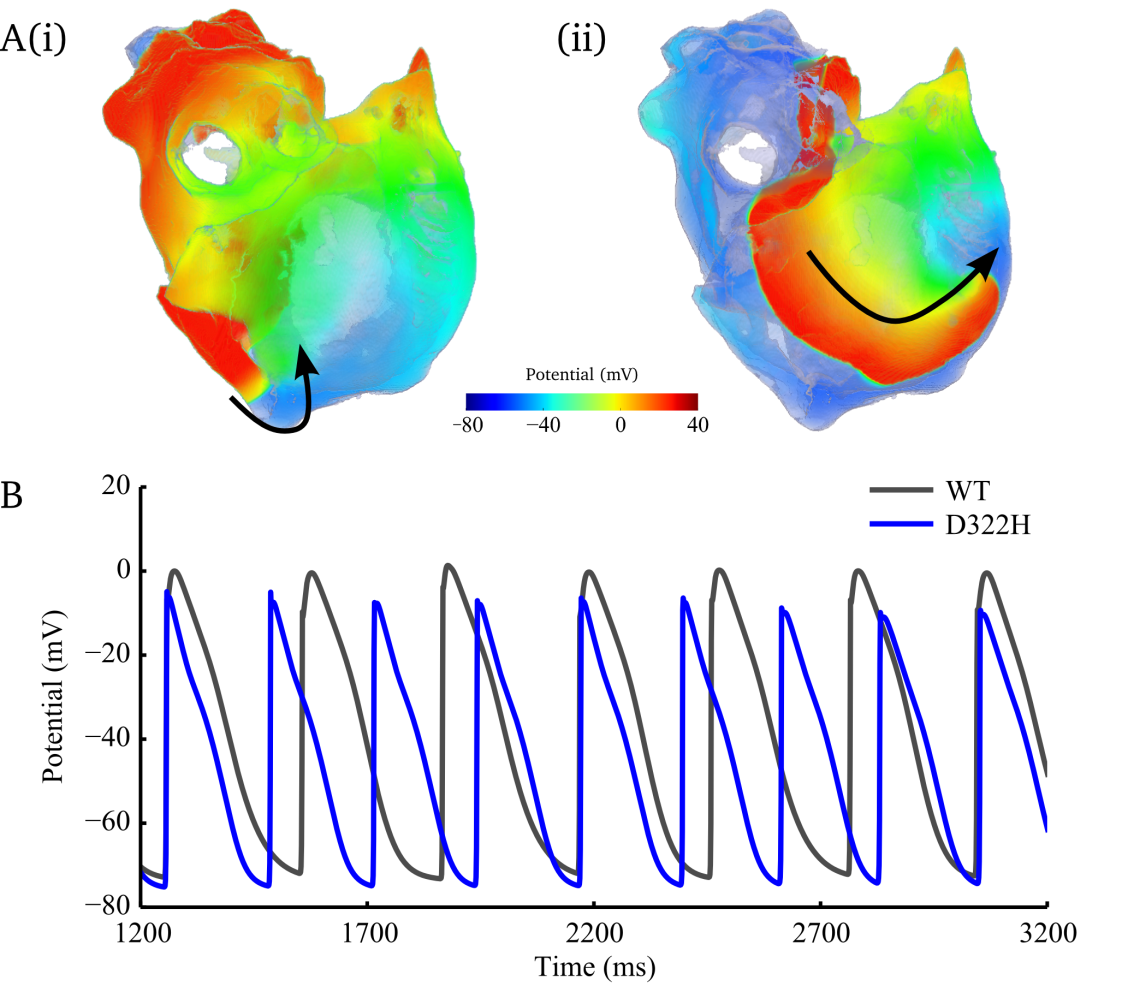


**Figure A** (A) Snapshots of simulated re-entrant excitation waves. (B) Simulated time course of activation potential during re-entrant excitation measured from a right atrial cell located close to the IVC. The action potential of myocytes carrying D322H was compared with the action potential of WT myocytes.

Shortening of the APD in tissue in D322H as compared to the WT is due to the electronic load compensating for the loss of terminal repolarising currents. Because D322H shortened the initial phase of the AP compared to wild type, when coupled the terminal prolongation is now inhibited due to the electrotonic load.

Table A shows the upper bound of ***D*** values sustaining re-entrant waves. ***D*** is the diffusion tensor parameter of the monodomain equation (presented in MS 5.3).

| Mutants | Threshold of ***D*** (% of control condition) |
| --- | --- |
| WT | 63% |
| A305T | 73% |
| D322H | 66% |
| E48G | 65% |
| D469E | 63% |
| Y155C | 64% |
| P488S | 36% |

**Table A** Mutations altered threshold of ***D*** values sustaining re-entrant waves. The value in the second column is the maximum value of ***D*** for which re-entrant excitation could be sustained (>10 s).

In simulations with Courtemanche *et al.* model, the loss-of-function mutation led to different behaviours in spiral wave excitation in tissue. Re-entry was terminated in the atria with Y155C and D469E, whereas the mutant P488S produced sustained re-entry within the time span of simulation with AP showing pronounced alternans (Figure B).


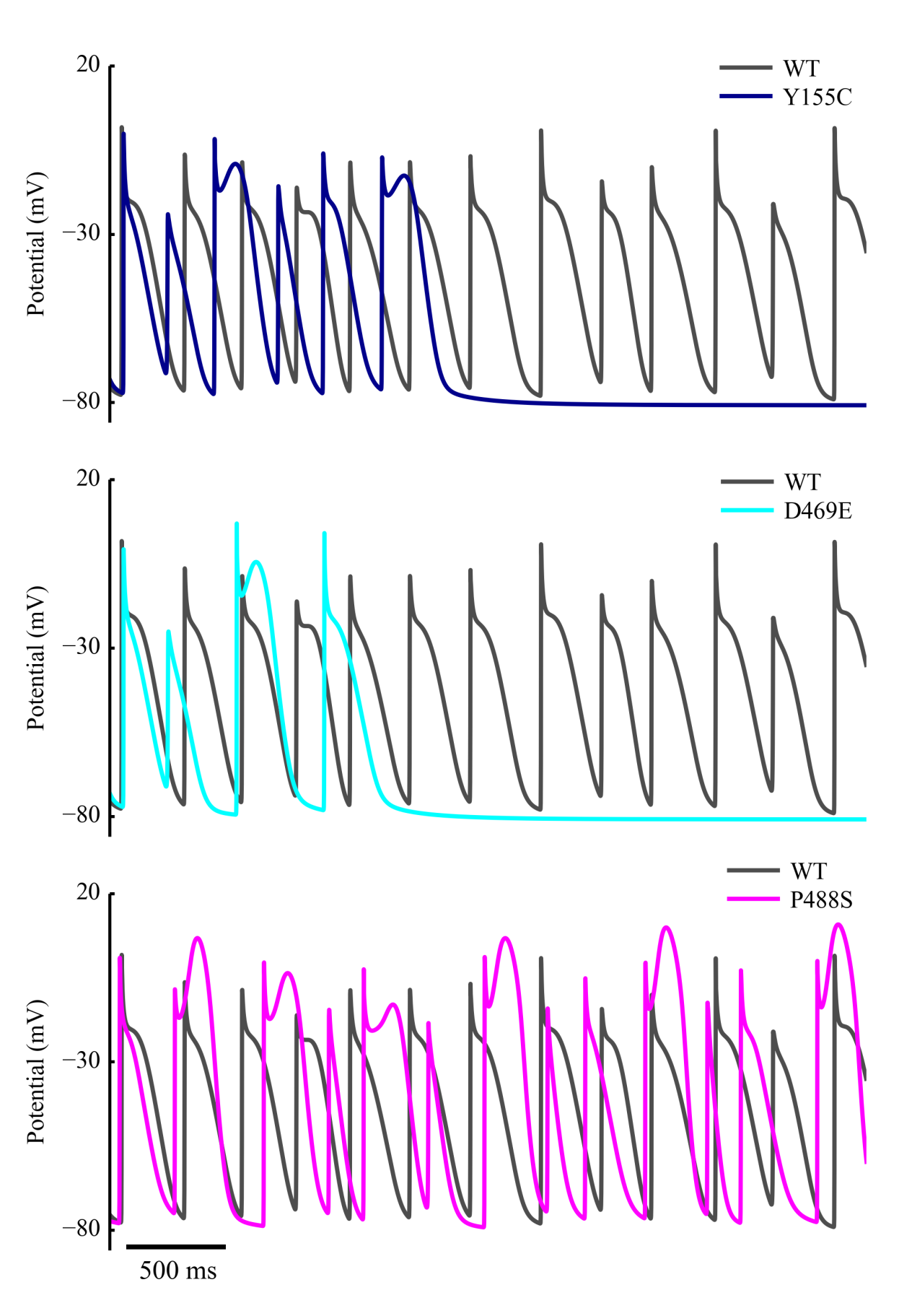


**Figure B** Time course of activation potentials for loss-of-function mutants recorded from a right atrial cell located close to the IVC in simulated re-entry using the *Courtmanche* et al. model.
