# Supplementary material for: In silico assessment of genetic variation in KCNA5 reveals multiple mechanisms of human atrial arrhythmogenesis
Source: PLoS Comput Biol. 2017 Jun 16;13(6):e1005587. doi: 10.1371/journal.pcbi.1005587 (PMC5493429; doi:10.1371/journal.pcbi.1005587)
Supplement: S4 Text — (DOCX) [file pcbi.1005587.s004.docx]

# Supporting Information 4: Vulnerability windows in the *Courtmanche et al*. model


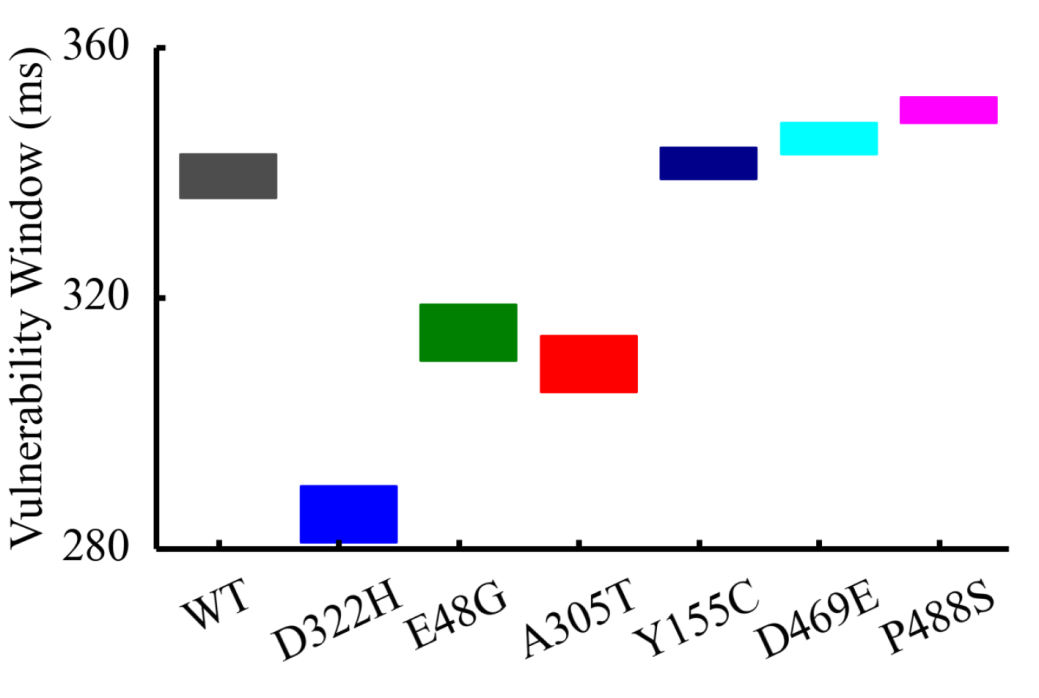


**Figure A.** The temporal vulnerability window in applying S2 stimulus towards generating excitation wave break at the CT/PM junction for mutations under lone AF conditions using the *Courtmanche et al.* model.
