# Supplementary material for: In silico assessment of genetic variation in KCNA5 reveals multiple mechanisms of human atrial arrhythmogenesis
Source: PLoS Comput Biol. 2017 Jun 16;13(6):e1005587. doi: 10.1371/journal.pcbi.1005587 (PMC5493429; doi:10.1371/journal.pcbi.1005587)
Supplement: S5 Text — (DOCX) [file pcbi.1005587.s005.docx]

# Supporting Information 5: Effects of beta-adrenergic stimulation on human atrial myocytes

Gain-of-function mutations demonstrated no enhanced vulnerability to the production of EADs compared to the wild type, as shown in Figure A.


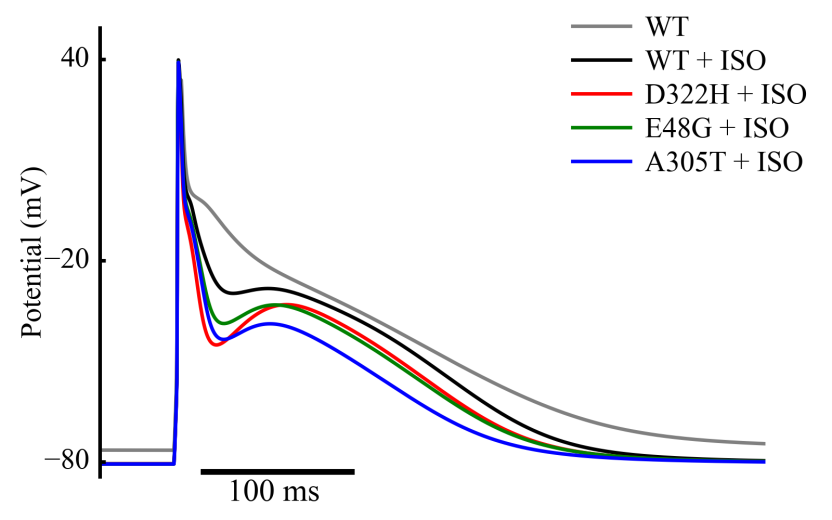


**Figure A** Applying ISO to human atrial myocytes did not induce APD prolongations versus control conditions or early-after-depolarisations with WT or gain-of-function mutants carrying I_Kur_.

Figure B illustrates simulated time courses of calcium transient (CaT), L-type calcium current (I_CaL_) and Na^+^-Ca^2+^ exchanger current (I_NaCa_) elicited by action potentials of RA cells for the loss-of-function mutations (left column) and CT/PM cells for the mutation D469E (right column) in the presence of ISO. For RA cells, pronounced EADs were observed for the mutations Y155C and P488S, while the APD was markedly prolonged by D469E (Figure B, Ai). Specifically, the loss-of-function mutation D469E induced EADs in CT cells but not in PM cells (Figure B, Aii). The CaT was increased by the loss-of-function mutations as compared to the WT (Figure B, Bi). Also, the CaT in CT cells was greater than that in PM cells (Figure B, Bii). In the presence of EADs, a significant reaction of I_CaL_ (Figure B, Ci-ii) and increased activity of I_NaCa_ (Figure B, Di-ii) were observed during late repolarisation phases of AP; a second peak in the CaT was also seen in these simulations.


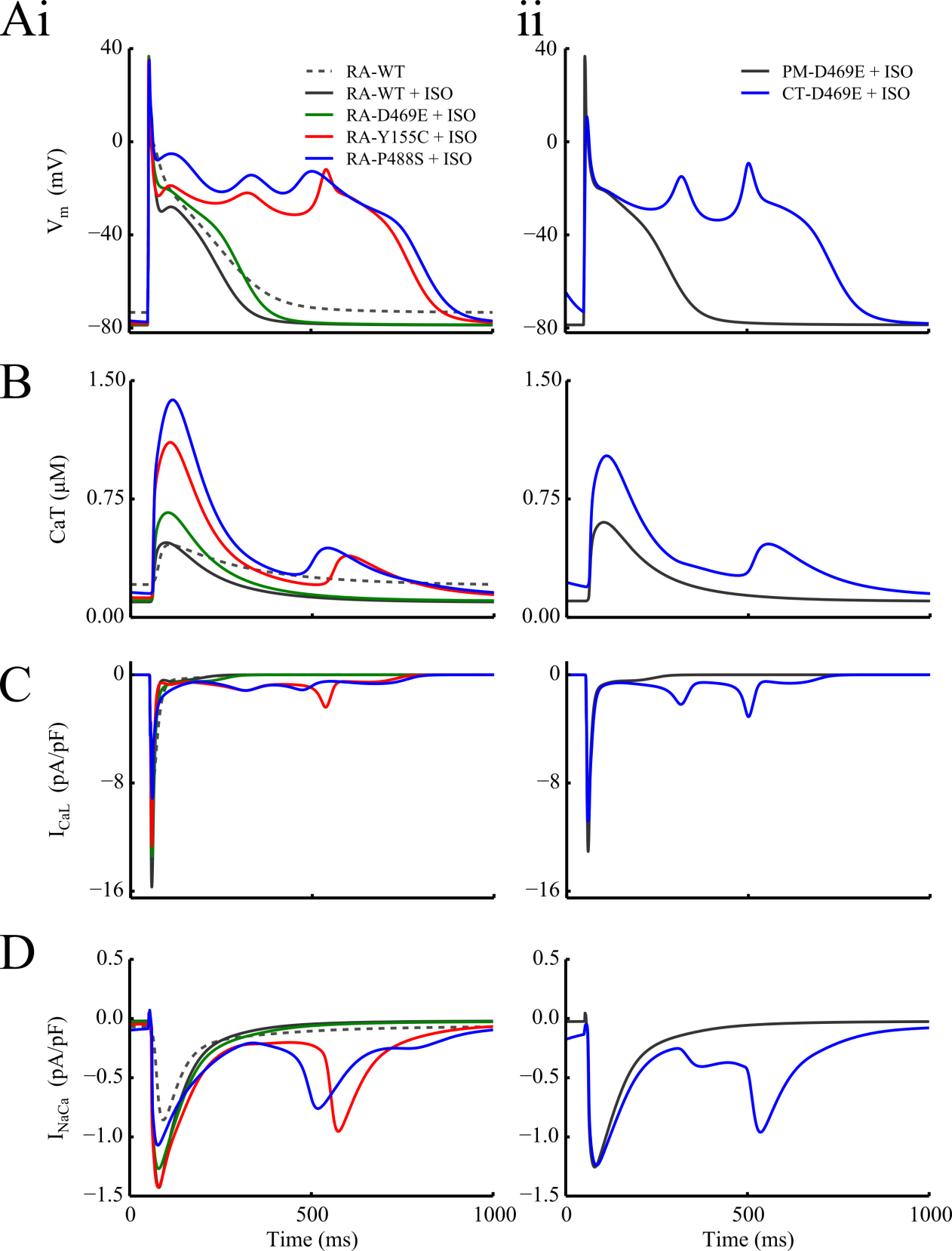


**Figure B** Effect of *KCNA5* loss-of-function mutations on the atrial action potential and the elicited calcium transient (CaT), L-type calcium (I_CaL_) and Na^+^-Ca^2+^ exchanger current (I_NaCa_) in the presence of ISO. **A** Action potential traces for the loss-function mutations and the WT: **(i)** RA cells; **(ii)** the CT and PM cells with the mutation D469E. **B-D** Time courses of **(B)** CaT, **(C)** I_CaL_ and **(D)** I_NaCa_ elicited during the action potentials shown in (A).

To test model dependency of single cell susceptibility to the induction of EADs in the loss of function mutations – observed only in the Grandi cell model – we further studied the potential to induce EADs in the Colman and CRN cell models in combination with the effects of Isoprenaline (ISO). However, EADs could not be induced in these cell models under reasonable modifications to parameters.

We also simulated ISO effects using Nygren *et al.* model for human atrial myocytes [1]. The effects of ISO on ionic currents were kept same to those simulated in the Grandi *et al.* model [2]. We show that Nygren *et al.* model produced pronounced EADs, which is consistent with the results from the Grandi *et al.* model (Figure C). Applying ISO to the atrial myocyte with 60% reduction in the channel conductance of *I*_Kur_ produced marked EADs.


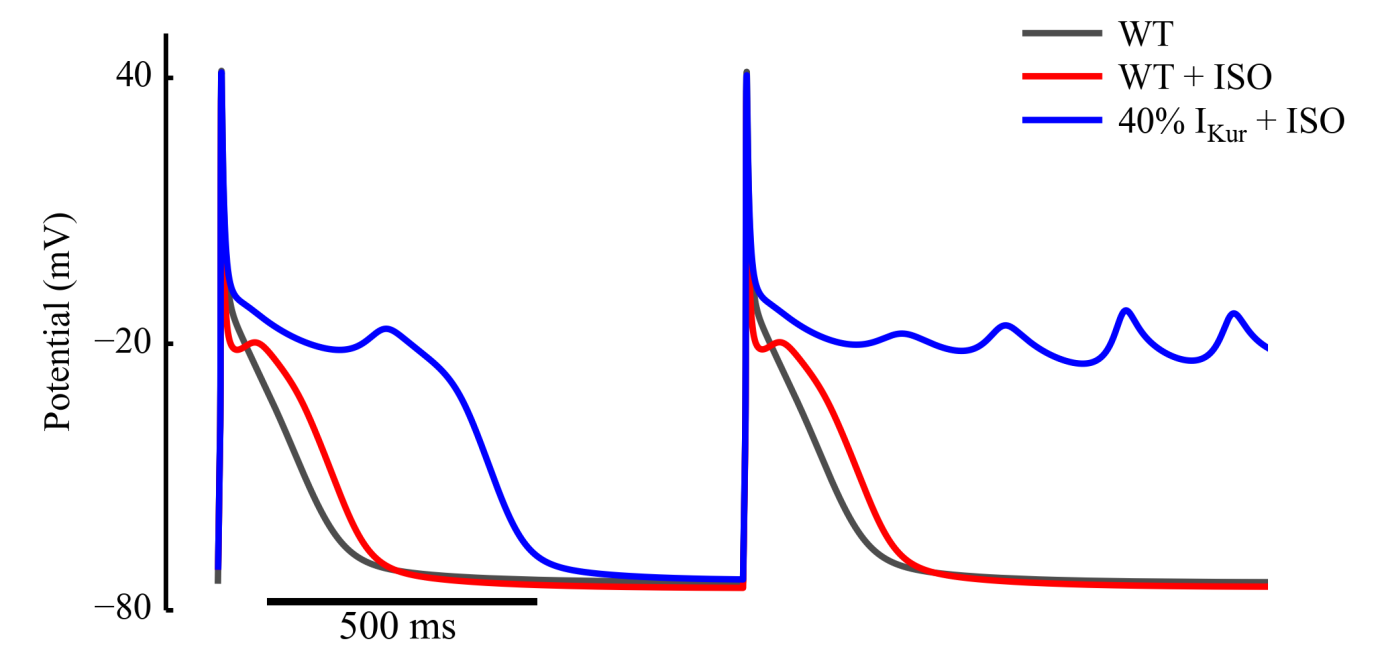


**Figure C** Simulated effects of ISO on human atrial AP using the Nygren *et al.* model. To model down-regulation in *I*_Kur_ by the loss-of-function mutations, the maximum conductance of *I*_sus_ in the Nygren *et al.* model was reduced to 40% of the original value.

**References**

1. Nygren A, Fiset C, Firek L, Clark JW, Lindblad DS, Clark RB, et al. Mathematical Model of an Adult Human Atrial Cell The Role of K+ Currents in Repolarization. Circ Res. 1998;82: 63–81. doi:10.1161/01.RES.82.1.63

2. Grandi E, Pandit SV, Voigt N, Workman AJ, Dobrev D, Jalife J, et al. Human Atrial Action Potential and Ca2+ Model Sinus Rhythm and Chronic Atrial Fibrillation. Circ Res. 2011;109: 1055–1066. doi:10.1161/CIRCRESAHA.111.253955
