# Supplementary material for: In silico assessment of genetic variation in KCNA5 reveals multiple mechanisms of human atrial arrhythmogenesis
Source: PLoS Comput Biol. 2017 Jun 16;13(6):e1005587. doi: 10.1371/journal.pcbi.1005587 (PMC5493429; doi:10.1371/journal.pcbi.1005587)
Supplement: S6 Text — (DOCX) [file pcbi.1005587.s006.docx]

# Supporting Information 6: Validations to the updated *Colman et al.* model with a new formulation of *I*_Kur_

Figure A shows the simulated rate-dependence of AP and APD using the updated Colman et al. model. A comparison of APD_90_ between the model and multiple experimental datasets is illustrated in Table A.


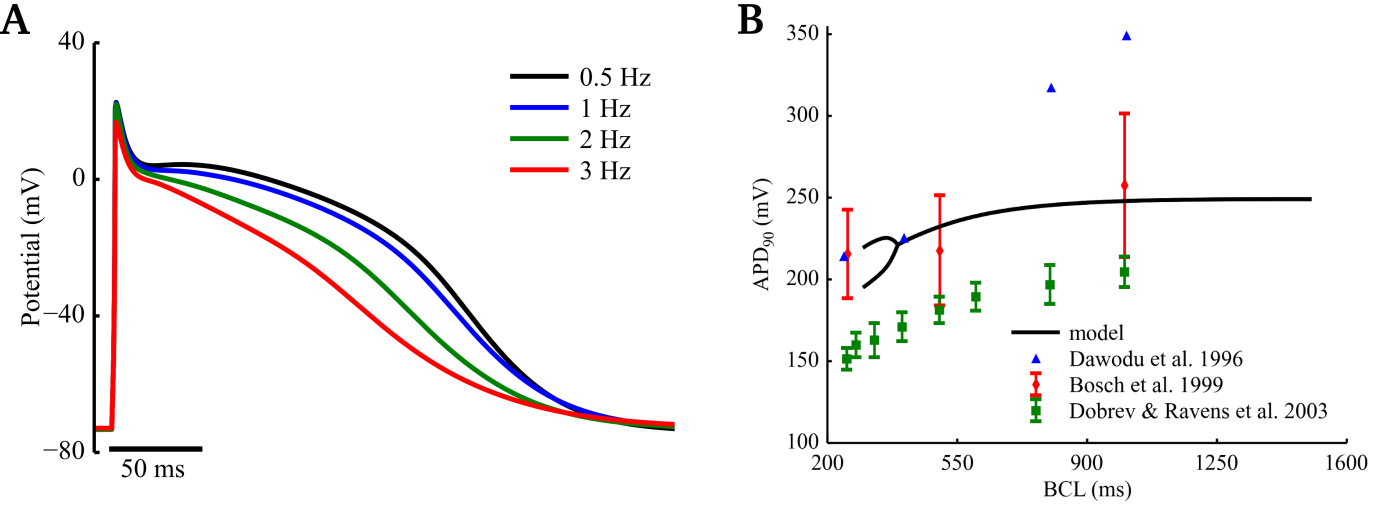


**Figure A** Rate dependence of simulated APs of the human right atrium. (A) APs produced with multiple stimulus rates. (B) APD restitution for the model, compared to a number of experimental data sets ([1–3]).

| **Study** | **APD_90_ (ms) (1 Hz)** |
| --- | --- |
| Model | 248 |
| Pau *et al.* 2007 [4] | 239 ± 11 |
| Redpath *et al.* 2006 [5] | 210 ± 17 |
| Katoh *et al.* 2005 [6] | 255 ± 39 |
| Dawodu *et al.* 1996 [7] | 361 ± 71 |
| Bosch *et al.* 1999 [2] | 255 ± 45 |
| Dobrev *et al.* 2002 [8] | 203 ± 10 |
| Kim *et al.* 2002 [9] | 258 ± 25 |
| Sanchez *et al.* 2014 [10] | 318 ± 42 |

**Table A** Validation of APD_90_ of the updated Colman *et al.* model of human atrial electrophysiology against experimental data.

**References**

1. Dawodu AA, Monti F, Iwashiro K, Schiariti M, Chiavarelli R, Puddu PE. The shape of human atrial action potential accounts for different frequency-related changes in vitro. Int J Cardiol. 1996;54: 237–249. doi:10.1016/0167-5273(96)02605-8

2. Bosch RF, Zeng X, Grammer JB, Popovic K, Mewis C, Kühlkamp V. Ionic mechanisms of electrical remodeling in human atrial fibrillation. Cardiovasc Res. 1999;44: 121–131. doi:10.1016/S0008-6363(99)00178-9

3. Dobrev D, Ravens U. Remodeling of cardiomyocyte ion channels in human atrial fibrillation. Basic Res Cardiol. 2003;98: 137–148. doi:10.1007/s00395-003-0409-8

4. Pau D, Workman AJ, Kane KA, Rankin AC. Electrophysiological and arrhythmogenic effects of 5-hydroxytryptamine on human atrial cells are reduced in atrial fibrillation. J Mol Cell Cardiol. 2007;42: 54–62. doi:10.1016/j.yjmcc.2006.08.007

5. Redpath CJ, Rankin AC, Kane KA, Workman AJ. Anti-adrenergic effects of endothelin on human atrial action potentials are potentially anti-arrhythmic. J Mol Cell Cardiol. 2006;40: 717–724. doi:10.1016/j.yjmcc.2006.01.012

6. Katoh H, Shinozaki T, Baba S, Satoh S, Kagaya Y, Watanabe J, et al. Monophasic action potential duration at the crista terminalis in patients with sinus node disease. Circ J Off J Jpn Circ Soc. 2005;69: 1361–1367.

7. Dawodu AA, Monti F, Iwashiro K, Schiariti M, Chiavarelli R, Puddu PE. The shape of human atrial action potential accounts for different frequency-related changes in vitro. Int J Cardiol. 1996;54: 237–249.

8. Dobrev D, Ravens U. Remodeling of cardiomyocyte ion channels in human atrial fibrillation. Basic Res Cardiol. 2003;98: 137–148. doi:10.1007/s00395-003-0409-8

9. Kim B-S, Kim Y-H, Hwang G-S, Pak H-N, Lee SC, Shim WJ, et al. Action potential duration restitution kinetics in human atrial fibrillation. J Am Coll Cardiol. 2002;39: 1329–1336. doi:10.1016/S0735-1097(02)01760-6

10. Sánchez C, Bueno-Orovio A, Wettwer E, Loose S, Simon J, Ravens U, et al. Inter-Subject Variability in Human Atrial Action Potential in Sinus Rhythm versus Chronic Atrial Fibrillation. PLoS ONE. 2014;9: e105897. doi:10.1371/journal.pone.0105897
