# Supplementary material for: In silico assessment of genetic variation in KCNA5 reveals multiple mechanisms of human atrial arrhythmogenesis
Source: PLoS Comput Biol. 2017 Jun 16;13(6):e1005587. doi: 10.1371/journal.pcbi.1005587 (PMC5493429; doi:10.1371/journal.pcbi.1005587)
Supplement: S7 Text — (DOCX) [file pcbi.1005587.s007.docx]

# Supporting Information 7: Regional cell models

Regional cell models are implemented based on previous modelling studies [1–3] utilizing both canine and human data. All regional cell models are derived from the baseline cell model (RA) by adjusting the current densities of various ion currents, according to the relative differences between different regions (Table A).

|  | **CT** | **BB** | **PM** | **AVR** | **RAA** | **AS** | **LA** | **LAA** | **PV** |
| --- | --- | --- | --- | --- | --- | --- | --- | --- | --- |
| **I_CaL_** | x1.68 | x1.72 | x0.94 | x0.67 | - | x0.4 | - |  | x0.7 |
| **I_to_** | x1.35 | x1.35 | - | x0.6 | x0.53 | x0.212 | - | x0.53 | x0.75 |
| **I_Kur_** | - | - | - | - | - | x0.667 | - | x0.8 | - |
| **I_Na_** | - | - | - | - | - | x1.3 | - |  | - |
| **I_Kr_** | - | - | - | x1.63 | - | - | x1.6 | x1.6 | x2.4 |
| **I_K1_** | - | - | - | - | - | V_1/2_ -6 | - |  | x0.62 |

**Table A.** Scaling factors for each current for various regions relative to the RA (baseline) cell models. CT = crista terminalis, BB = Bachmann’s bundle, PM = pectinate muscles, AVR = atrio-ventricular ring, RAA = right atrial appendage, AS = atrial septum, LA = left atrium, LAA = left atrial appendage, PV = pulmonary veins.

These parameters were used in the *Colman* *et al.* and *Courtemanche* *et al.* cell models to produce the family of regional cell models (Figure A).

For the purposes of investigation into the behaviour of EADs in the right atrium, cell models were derived for the CT and PM for the *Grandi* *et al.* model using the parameters in Table A (Figure B). For this implementation, the conductance of *I_CaL_* in the CT was increased by a factor of 1.2 only as EADs were produced in normal conditions at the full 1.68 scaling of *I*_CaL_.


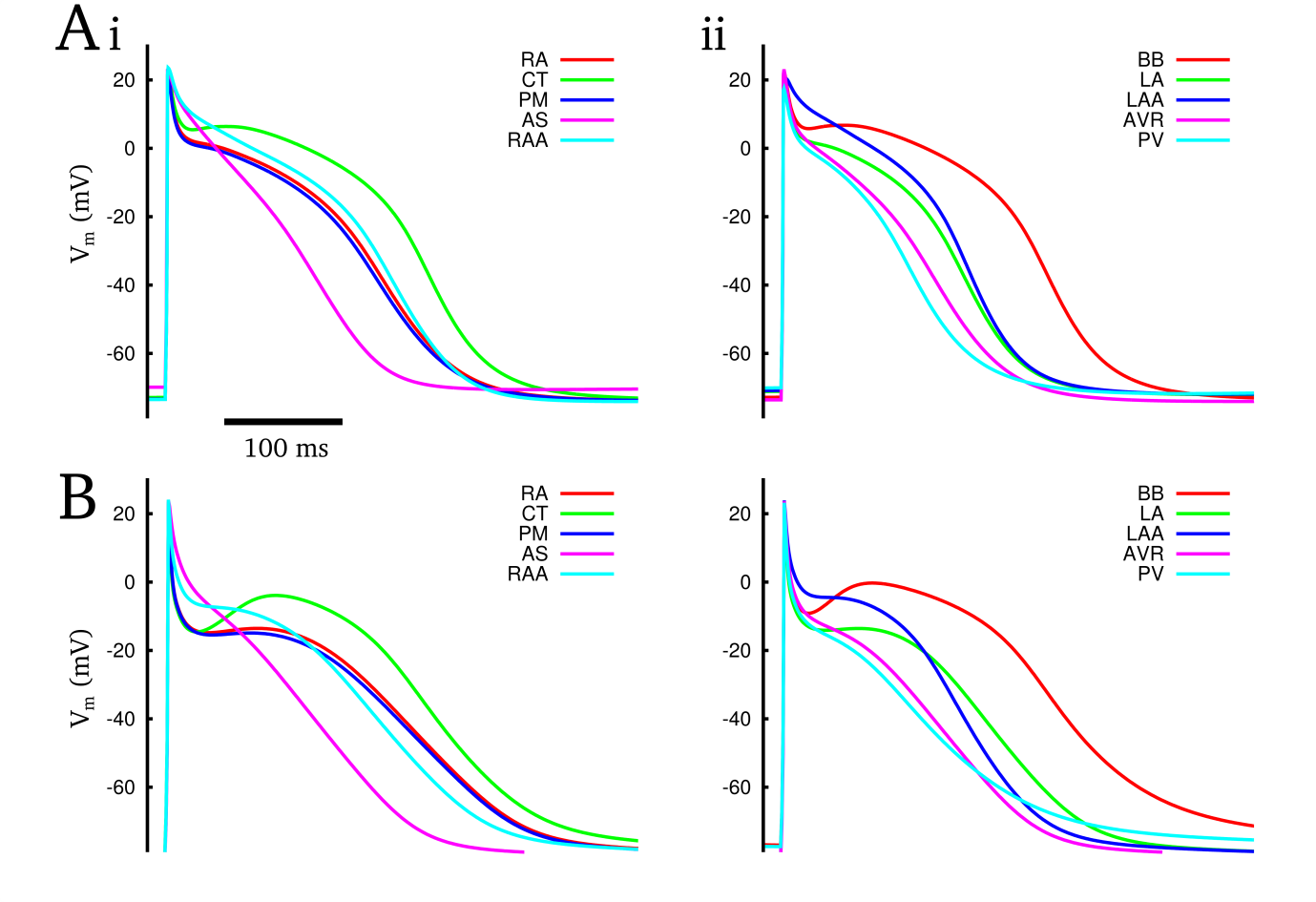


**Figure A** Regional cell model APs in the *Colman* *et al.* (A) and *Courtemanche* *et al.* (B) cell models, for regions within the right (i) and left (ii) atria at a cycle length of 1000 ms. Abbreviations are the same as those in the legend for Table SI7.1.


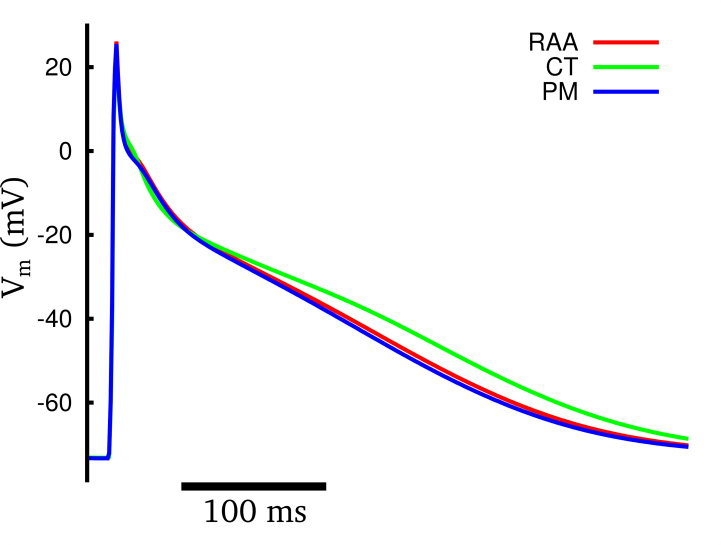


**Figure B.** Cell models for the CT and PM in *Grandi* *et al.* model, compared to its baseline (RAA). Note that the morphology is substantially different to the other models – this is primarily due to the triangular morphology of the baseline model.

**References**

1. Seemann G, Höper C, Sachse FB, Dössel O, Holden AV, Zhang H. Heterogeneous three-dimensional anatomical and electrophysiological model of human atria. Philos Transact A Math Phys Eng Sci. 2006;364: 1465–1481. doi:10.1098/rsta.2006.1781

2. Dorn A, Krueger MW, Seemann G, Doessel O. Modelling of heterogeneous human atrial electrophysiology. Biomed Tech (Berl). 2012;57 Suppl 1. doi:10.1515/bmt-2012-4215

3. Colman MA, Aslanidi OV, Kharche S, Boyett MR, Garratt C, Hancox JC, et al. Pro-arrhythmogenic Effects of Atrial Fibrillation Induced Electrical Remodelling- Insights from the 3D Virtual Human Atria. J Physiol. 2013;591: 4249–4272. doi:10.1113/jphysiol.2013.254987
