# Supplementary material for: In silico assessment of genetic variation in KCNA5 reveals multiple mechanisms of human atrial arrhythmogenesis
Source: PLoS Comput Biol. 2017 Jun 16;13(6):e1005587. doi: 10.1371/journal.pcbi.1005587 (PMC5493429; doi:10.1371/journal.pcbi.1005587)
Supplement: S8 Text — (DOCX) [file pcbi.1005587.s008.docx]

# Supporting Information 8: Modelling chronic AF induced electrical remodelling

Chronic AF (cAF) is associated with electrical remodelling of multiple ion channel currents [1], resulting in a significantly shortened APD [1–3]. cAF electrical remodelling is incorporated into the human atrial cell models through modifications to the conductance and kinetics of remodelled ion channel currents according to previous publications: In the *Colman* *et al.* and *Courtemanche et al. models*, parameters are taken from our previous publication [4]; remodelling is incorporated into the *Grandi et al.* cell model according to the original study. Modifications to *I_Kur_* associated with electrical remodelling are not included, such that the form of *I_Kur_* is given by the mutants only. Parameter modifications are shown in Table A.

In tissue models, the diffusion coefficient ***D*** was reduced by 50% to model the remodelling in gap junctions and structural remodelling under cAF conditions [2].

| Substrate | *Colman et al. model*  *Courtemanche et al. model* | *Grandi et al. model* |
| --- | --- | --- |
| I_Na_ | *No change* | *-10%* |
| I_NaL_ | *N.A* | *Added INaL* |
| I_CaL_ | -70% | -50% |
| I_Kur_ | -50% | -55% |
| I_to_ | -65% | -80% |
| I_K1_ | +100% | +100% |
| I_Ks_ | +100% | +100% |
| I_NaCa_ | +55% | +40% |
| I_Kr_ | No change | No change |
| SERCA | +50% | Reduced maximum pump rate |
| RyR | +300% | Ca^2+^ sensitivity increased by 2-fold |
| SR Ca^2+^ leak | +25% | +25% |

**Table A.** Modifications to the model parameters in simulating the cAF conditions versus SR conditions for the three models; parameters are taken from [2,3].

**References**

1. Bosch RF, Zeng X, Grammer JB, Popovic K, Mewis C, Kühlkamp V. Ionic mechanisms of electrical remodeling in human atrial fibrillation. Cardiovasc Res. 1999;44: 121–131. doi:10.1016/S0008-6363(99)00178-9

2. Colman MA, Aslanidi OV, Kharche S, Boyett MR, Garratt C, Hancox JC, et al. Pro-arrhythmogenic effects of atrial fibrillation-induced electrical remodelling: insights from the three-dimensional virtual human atria. J Physiol. 2013;591: 4249–4272. doi:10.1113/jphysiol.2013.254987

3. Grandi E, Pandit SV, Voigt N, Workman AJ, Dobrev D, Jalife J, et al. Human Atrial Action Potential and Ca2+ Model Sinus Rhythm and Chronic Atrial Fibrillation. Circ Res. 2011;109: 1055–1066. doi:10.1161/CIRCRESAHA.111.253955

4. Colman MA, Aslanidi OV, Kharche S, Boyett MR, Garratt C, Hancox JC, et al. Pro-arrhythmogenic Effects of Atrial Fibrillation Induced Electrical Remodelling- Insights from the 3D Virtual Human Atria. J Physiol. 2013;591: 4249–4272. doi:10.1113/jphysiol.2013.254987
