# Supplementary material for: In silico assessment of genetic variation in KCNA5 reveals multiple mechanisms of human atrial arrhythmogenesis
Source: PLoS Comput Biol. 2017 Jun 16;13(6):e1005587. doi: 10.1371/journal.pcbi.1005587 (PMC5493429; doi:10.1371/journal.pcbi.1005587)
Supplement: S9 Text — (DOCX) [file pcbi.1005587.s009.docx]

# Supporting Information 9: Human SAN model

Figure A illustrates simulated SAN AP, which is compared to an experimental SAN AP trace.

The in tissue SAN model accounts for heterogeneity in the SAN electrophysiology from the centre to the periphery (Figure B) using a continuous gradient [1,2]. ISO is also implemented in the SAN model based on our previous model (which can be found in [3] but has not been published elsewhere). The spontaneous pacing rate of the SAN in single cell and in tissue under both baseline and ISO conditions are consistent with experimental evidence (~ 1Hz in control [4] and ~1.4 Hz under ISO [5]). Activation time of the SAN is ~ 80 ms [6] with conduction velocity of ~ 0.06 m/s near the SAN centre, increasing gradually to the atrial value at the periphery.


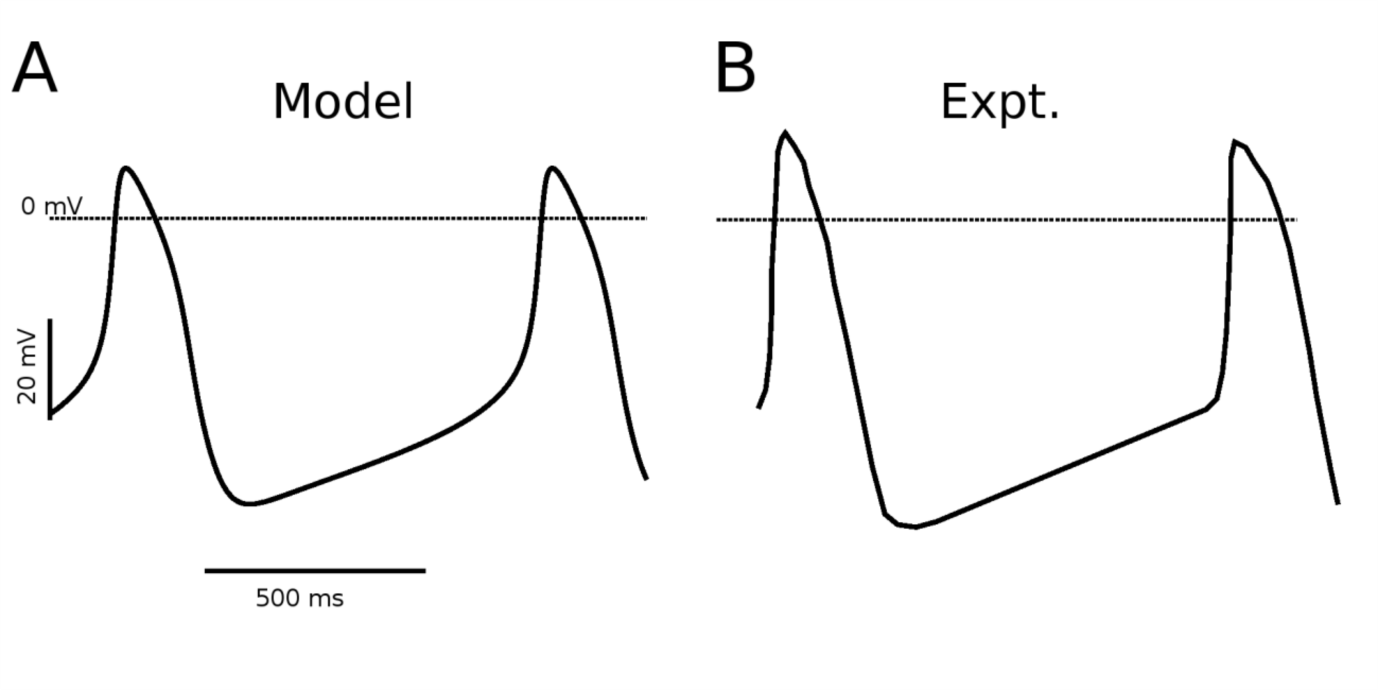


**Figure A** Simulated AP of human SAN as compared to experimental traces, which is extracted from [7].


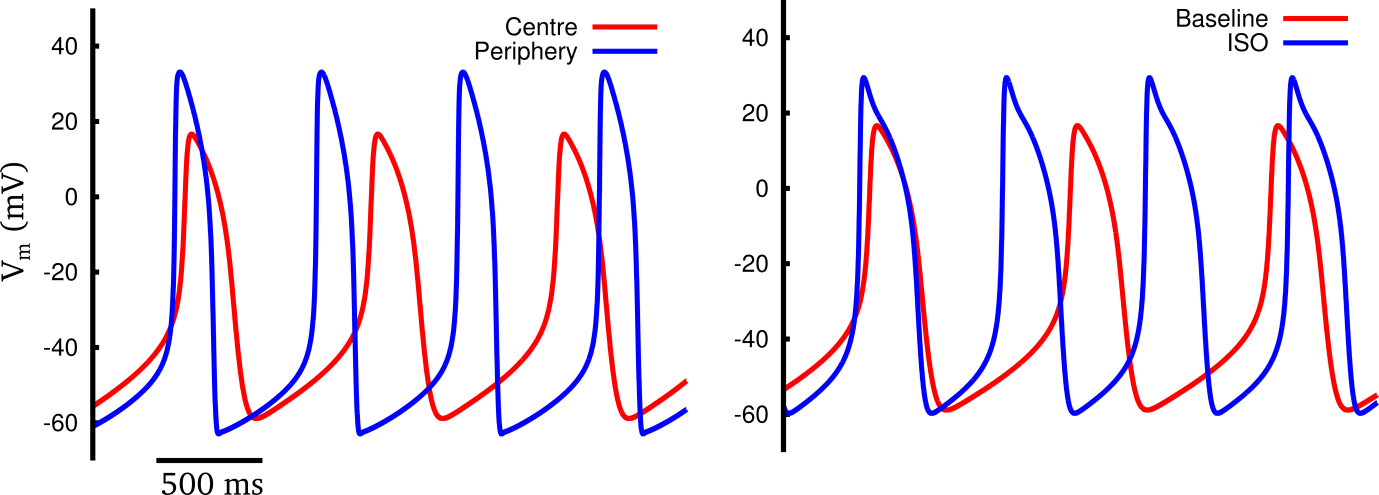


**Figure B** Human SAN models in the centre and periphery (left) and for the centre, under baseline and ISO conditions (right).

**References**

1. Seemann G, Höper C, Sachse FB, Dössel O, Holden AV, Zhang H. Heterogeneous three-dimensional anatomical and electrophysiological model of human atria. Philos Transact A Math Phys Eng Sci. 2006;364: 1465–1481. doi:10.1098/rsta.2006.1781

2. Colman MA. 3D Anatomical Modelling of the Atria. Mechanisms of Atrial Arrhythmias. Springer International Publishing; 2014. pp. 159–184. Available: http://link.springer.com/chapter/10.1007/978-3-319-01643-6_6

3. Colman MA. Autonomic Regulation and Pathophysiological Remodelling. Mechanisms of Atrial Arrhythmias. Springer International Publishing; 2014. pp. 115–155. Available: http://link.springer.com/chapter/10.1007/978-3-319-01643-6_5

4. Verkerk AO, van Ginneken ACG, Wilders R. Pacemaker activity of the human sinoatrial node: role of the hyperpolarization-activated current, I(f). Int J Cardiol. 2009;132: 318–336. doi:10.1016/j.ijcard.2008.12.196

5. Fedorov VV, Chang R, Glukhov AV, Kostecki G, Janks D, Schuessler RB, et al. Complex interactions between the sinoatrial node and atrium during reentrant arrhythmias in the canine heart. Circulation. 2010;122: 782–789. doi:10.1161/CIRCULATIONAHA.109.935288

6. Fedorov VV, Glukhov AV, Chang R, Kostecki G, Aferol H, Hucker WJ, et al. Optical mapping of the isolated coronary-perfused human sinus node. J Am Coll Cardiol. 2010;56: 1386–1394. doi:10.1016/j.jacc.2010.03.098

7. Verkerk AO, Wilders R, Borren MMGJ van, Peters RJG, Broekhuis E, Lam K, et al. Pacemaker current (If) in the human sinoatrial node. Eur Heart J. 2007;28: 2472–2478. doi:10.1093/eurheartj/ehm339
