# Supplementary material for: In silico assessment of genetic variation in KCNA5 reveals multiple mechanisms of human atrial arrhythmogenesis
Source: PLoS Comput Biol. 2017 Jun 16;13(6):e1005587. doi: 10.1371/journal.pcbi.1005587 (PMC5493429; doi:10.1371/journal.pcbi.1005587)
Supplement: S10 Text — (DOCX) [file pcbi.1005587.s010.docx]

# Supporting Information 10: 3D anatomical model of the human atria

The anatomical atrial model is derived from the visible human dataset [1] and segmented into the major differentiated anatomical regions [1,2]. A reconstruction of the SAN [3] was previously incorporated into the model (Figure A, Ai-ii). It successfully reproduces normal atrial activation patterns observed in the human atria (Figure A, Bi-ii), with conduction velocities of 1.3 m/s in CT and 0.7 m/s in RA, in accordance with experimental values [2]. These velocities are reduced to 0.8 and 0.46 m/s in the most severe remodelling case (40 % ***D***). In the 3D simulations, the atria were paced from the SAN region (shown in red in Figure A, Ai).


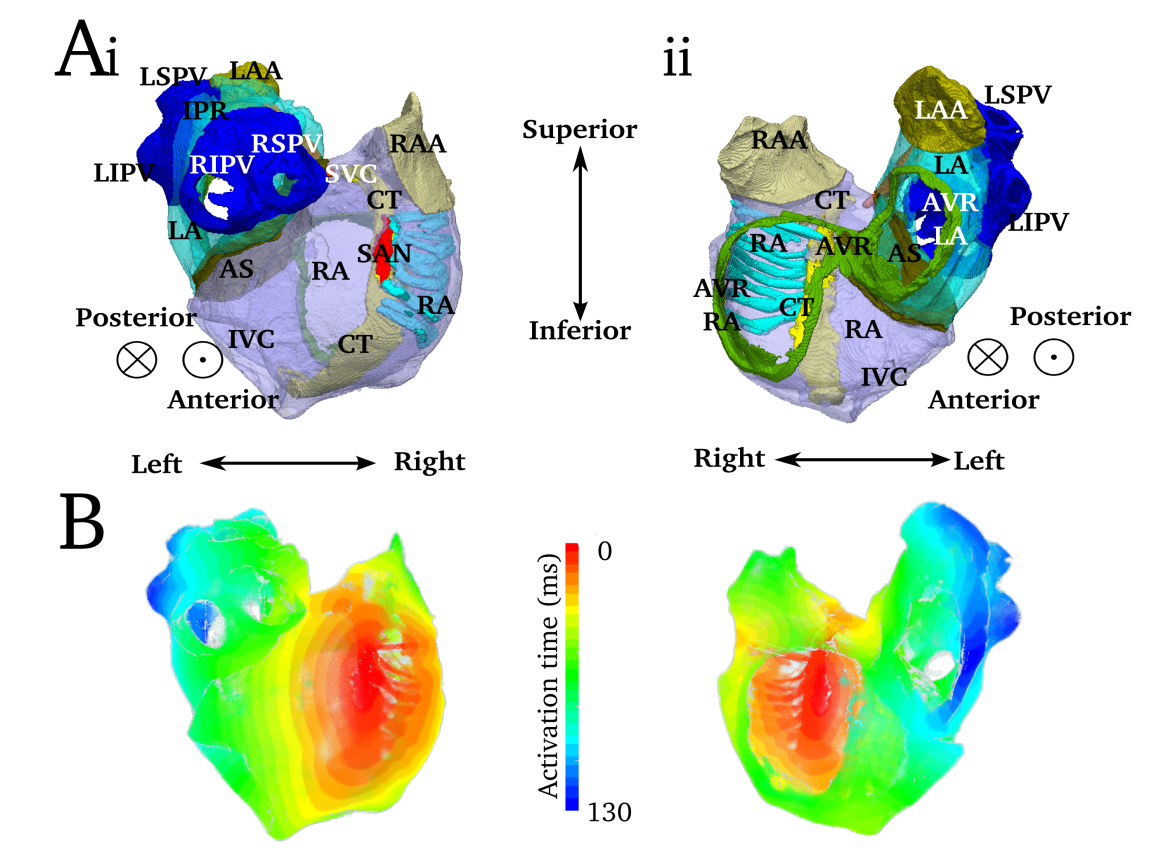


**Figure A.** Segmented anatomical reconstruction of the human atria (A) and the activation pattern under sinus rhythm (B) from two different views (i,ii). RA = right atrium, LA = left atrium, PV = pulmonary vein (Right/Left and Inferior/Superior), IPR = inter pulmonary region, LAA/RAA = left/right atrial appendage, SVC/IVC = superior/inferior vena cava, AVR = atrio-ventricular ring, CT = crista terminalis, SAN = sino-atrial node, AS = atrial septum.

**Reference**

1. Seemann G, Höper C, Sachse FB, Dössel O, Holden AV, Zhang H. Heterogeneous three-dimensional anatomical and electrophysiological model of human atria. Philos Transact A Math Phys Eng Sci. 2006;364: 1465–1481. doi:10.1098/rsta.2006.1781

2. Colman MA, Aslanidi OV, Kharche S, Boyett MR, Garratt C, Hancox JC, et al. Pro-arrhythmogenic Effects of Atrial Fibrillation Induced Electrical Remodelling- Insights from the 3D Virtual Human Atria. J Physiol. 2013;591: 4249–4272. doi:10.1113/jphysiol.2013.254987

3. Chandler N, Aslanidi O, Buckley D, Inada S, Birchall S, Atkinson A, et al. Computer three-dimensional anatomical reconstruction of the human sinus node and a novel paranodal area. Anat Rec Hoboken NJ 2007. 2011;294: 970–979. doi:10.1002/ar.21379
