# Supplementary material for: In silico assessment of genetic variation in KCNA5 reveals multiple mechanisms of human atrial arrhythmogenesis
Source: PLoS Comput Biol. 2017 Jun 16;13(6):e1005587. doi: 10.1371/journal.pcbi.1005587 (PMC5493429; doi:10.1371/journal.pcbi.1005587)
Supplement: S11 Text — (DOCX) [file pcbi.1005587.s011.docx]

# Supporting Information 11: Numerical methods

For the Colman *et al.* and Courtemanche *et al.* model, the gating variables were solved using the Rush-Larsen scheme [1], and the Forward Euler scheme was used for the remaining ordinary differential equations (ODEs). The Grandi *et al.* model was solved using CVODE [2], a free C package for solving initial value problems for stiff ODE system. All models were implemented using C/C++. For each model, a time step of 0.005 ms was used for the former two models, and 0.01 ms for the Grandi *et al.* model.

The current stimulation strength and duration were kept the same with the values used in the original models, respectively. The initial conditions of single cell models were obtained by pacing the model at 1 Hz until steady-state is reached, i.e. the change in beat-to-beat APD_90_ is less than 0.1%. Specially, for the Grandi *et al.* model, the initial conditions were obtained after the cellular model was paced by 100 times at 1Hz due to a small but constant variation in the beat-to-beat APD_90_. The acquired initial conditions of each model were used in the subsequent single cell simulations. To obtain the AP traces and APD values presented in MS Figure 1 and 2, each model were paced by 100 times before the values were recorded.

In simulations with 1D strand models, initial conditions were obtained from single cell models paced at a same pacing rate. In initiating the re-entry in the whole atria model, the initial conditions were produced from 1D strand simulations paced at 3 Hz with the same mutations/WT and under the same conditions (lone AF/chronic AF).

For the whole atria model, the tissue was discretised into regular grids with a spatial interval of 0.33 mm and the monodomain equation was solved using finite different scheme.

**References**

1. Rush S, Larsen H. A Practical Algorithm for Solving Dynamic Membrane Equations. IEEE Trans Biomed Eng. 1978;BME-25: 389–392. doi:10.1109/TBME.1978.326270

2. Cohen S, Alan, Hindmarsh C. Cvode, A Stiff/nonstiff Ode Solver In C. in C Computers in Physics. 1996. pp. 138–143.
